# An eight-miRNA signature as a potential biomarker for predicting survival in lung adenocarcinoma

**DOI:** 10.1186/1479-5876-12-159

**Published:** 2014-06-04

**Authors:** Xuelian Li, Yunrui Shi, Zhihua Yin, Xiaoxia Xue, Baosen Zhou

**Affiliations:** 1 Department of Epidemiology, School of Public Health, China Medical University, No.92 Beier Road, Heping District, Shenyang, 110001, Liaoning Province, PR China; 2 The First Hospital of China Medical University, No.155 Nanjing Street, Heping District, Shenyang 110001, Liaoning Province, PR China

**Keywords:** Lung adenocarcinoma, MicroRNA, Prognostic markers, Overall survival

## Abstract

**Background:**

Lung adenocarcinoma is a heterogernous disease that creates challenges for classification and management. The purpose of this study is to identify specific miRNA markers closely associated with the survival of LUAD patients from a large dataset of significantly altered miRNAs, and to assess the prognostic value of this miRNA expression profile for OS in patients with LUAD.

**Methods:**

We obtained miRNA expression profiles and corresponding clinical information for 372 LUAD patients from The Cancer Genome Atlas (TCGA), and identified the most significantly altered miRNAs between tumor and normal samples. Using survival analysis and supervised principal components method, we identified an eight-miRNA signature for the prediction of overall survival (OS) of LUAD patients. The relationship between OS and the identified miRNA signature was self-validated in the TCGA cohort (randomly classified into two subgroups: n = 186 for the training set and n = 186 for the testing set). Survival receiver operating characteristic (ROC) analysis was used to assess the performance of survival prediction. The biological relevance of putative miRNA targets was also analyzed using bioinformatics.

**Results:**

Sixteen of the 111 most significantly altered miRNAs were associated with OS across different clinical subclasses of the TCGA-derived LUAD cohort. A linear prognostic model of eight miRNAs (miR-31, miR-196b, miR-766, miR-519a-1, miR-375, miR-187, miR-331 and miR-101-1) was constructed and weighted by the importance scores from the supervised principal component method to divide patients into high- and low-risk groups. Patients assigned to the high-risk group exhibited poor OS compared with patients in the low-risk group (hazard ratio [HR] = 1.99, *P* <0.001). The eight-miRNA signature is an independent prognostic marker of OS of LUAD patients and demonstrates good performance for predicting 5-year OS (Area Under the respective ROC Curves [AUC] = 0.626, *P* = 0.003), especially for non-smokers (AUC = 0.686, *P* = 0.023).

**Conclusions:**

We identified an eight-miRNA signature that is prognostic of LUAD. The miRNA signature, if validated in other prospective studies, may have important implications in clinical practice, in particular identifying a subgroup of patients with LUAD who are at high risk of mortality.

## Background

Lung adenocarcinoma (LUAD), is the most common histological subtype of non-small cell lung cancer (NSCLC) in females (smokers or non-smokers), and in non-smoking males. The incidence of LUAD has increased markedly over the past few decades in many countries, including China [1,2]. Most adenocarcinomas first occur in the outer region of the lungs with a tendency to spread to the lymph nodes and beyond. Despite advances in diagnosis and treatment, lung cancer mortality has increased. Mortality rates are amongst the highest of any cancer type.

Following advances in genomics, proteomics and molecular pathology, many candidate biomarkers with potential clinical value have been identified [3]. Further development of genomic biomarkers is expected to improve patient stratification and lead to more personalized treatment. MicroRNAs (miRNAs, miRs) are small, non-coding RNAs of 18–25 nucleotides, and are thought to regulate gene expression post-transcriptionally by causing mRNA degradation and/or repressing mRNA translation [4]. MiRNAs are frequently dysregulated in cancer, and may function as both oncogenes and tumor suppressors [4,5]. Several prognostic and predictive miRNA markers have been identified for NSCLC [6-11]. However, owing to the small datasets used, the heterogeneous nature of the disease and pre-selection of miRNAs and variations in the approaches for data pre-processing, there are inconsistencies in these sets of miRNA markers.

The purpose of this study is to identify specific miRNA markers closely associated with the survival of LUAD patients from a large dataset of significantly altered miRNAs, and to assess the prognostic value of this miRNA expression profile for OS in patients with LUAD.

## Methods

### TCGA miRNA dataset and patient information

MiRNA expression data and corresponding clinical data for 448 LUAD patients were obtained from The Cancer Genome Atlas (TCGA) data portal (January 2013) [12]. Both the miRNA expression data and clinical data, including outcome and staging information of TCGA LUAD patients deposited at the Data Coordinating Center (DCC), are publically available and open-access. TCGA data are classified by data type (clinical, mutations, gene expression) and data level, to allow structured access to this resource with appropriate patient privacy protection. This study meets the publication guidelines provided by TCGA [13]. The expression of 1046 human miRNAs in LUAD samples was assessed using the Illumina HiSeq Systems (n = 385) and Genome Analyzer (n = 63). MiRNA expression profiles for normal lung tissues (n = 46) were also analyzed using the Illumina HiSeq System. Level 3, normalized miRNA expression data (the calculated expression for all reads aligning to a particular miRNA per sample) were collected from the TCGA Data Portal using the Data Browser tool and quantile normalized [14], before performing downstream analysis. Samples and corresponding clinical data were cross-referenced by tumor barcodes. Owing to possible unrelated causes of death, 76 patients with an overall survival (OS) of less than 1 month were removed from the analysis. A total of 372 LUAD patients, including 196 females (mean age 66.23 ± 9.44 years) and 176 males (mean age 65.69 ± 9.94 years), were enrolled in the study (median follow-up: 15.23 months). To validate the miRNA markers being a specific signature or panel for LUAD, the data of TCGA lung squamous cell carcinoma (lung SCC), (321 patients) were also downloaded.

### Identification of differentially expressed miRNAs in LUAD and normal lung tissue samples

To identify miRNAs differentially expressed between LUAD and normal lung tissues, the raw counts of TCGA miRNA expression (level 3 data) obtained from the TCGA dataset (Illumina HiSeq Systems,385 LUAD samples and 46 normal controls) were normalized by a weighted trimmed mean of the log expression ratios (Trimmed mean of M values method, TMM) [15] using the R/Bioconductor package of edgeR [16]. Since many miRNAs were not expressed in certain tissue types or showed little variation over the patients in the dataset, only miRNAs expressed in at least two normal or tumor samples, with at least 100 counts per million were retained in the profile. A generalized linear model (GLM) was used to remove the batch effect. The expression differences were characterized by logFC (log 2 fold change) and associated *P*- values. LogFC indicates the fold change in expression of each miRNA from LUAD to normal lung tissue. Down- and up-regulated miRNAs were assigned a logFC < -1 and logFC >1 respectively, with FDR-adjusted *P* < 0.05.

### Survival analysis

A univariate Cox model was used to investigate the relationship between the continuous expression level of each miRNA and OS within different independent classes of disease stage, lymph node involvement (N stage), neoplasm metastasis (M stage), and size of original (primary) tumor (T stage). The Kaplan-Meier and log-rank method (Mantel-Haenszel test) were performed to test the equality for survival distributions in different groups. Hazard ratios (HRs), the ratio of hazards for a 2-fold change in the gene expression level, from univariate Cox regression analysis were used to identify candidate miRNAs associated with OS. MiRNAs with a HR < 1 were defined as a protective signature and those with HR for death > 1 were defined as high-risk miRNAs. The Cox proportional hazard model was used for multivariate analysis to identify miRNAs profiles or covariates with independent prognostic value.

### Definition of prognostic model and ROC curve

Univariate survival analyses were used to identify common miRNA related to OS within each of the following independent classes: disease stage, N stage, M stage and T stage. Within each group of clinical characteristics, the patient subclasses represented non-overlapping sets. Common miRNAs associated with OS in at least two independent categories for each covariate were selected as candidate markers, using a *P*-value of 0.1 as the cutoff for miRNA selection. The self-validated method (186 randomly selected samples as the training set and the other 186 samples as the validated set) was used to develop a prognostic model of the weighted linear combination of the detected miRNA expression levels. This algorithm is based on an importance score assigned to each miRNA, calculated by the supervised principal components method [17] and using the 10-fold cross-validation for selection of significant miRNAs. The prognostic score was calculated as follows: Prognostic-score = (0.181 × expression level of miR-31) + (0.136 × expression level of miR-196b) + (-0.114 × expression level of miR-375) + (-0.148 × expression level of miR-187) + (-0.352× expression level of miR-331) + (-0.372× expression level of miR-101-1) + (0.182× expression level of miR-766) + (0.21× expression level of miR-519a-1).

We used the linear miRNA prognostic model obtained from the training set to calculate an eight-miRNA signature prognostic score for each of the 372 patients. From the eight-miRNA signature prognostic scores we classified the samples into high-risk or low-risk group using the median score from the training set as a cutoff. Kaplan-Meier survival curves for the cases predicted to have low or high risk were generated. The prognostic performance was measured using time-dependent receiver operating characteristic (ROC) curves [18] by comparing the area under the respective ROC curves (AUC). Since the majority of events occurred before 60 months, the ability of models to predict outcome at and around 60 months was assessed. Permutation *P*-values of AUC were calculated from 1000 permutations of the survival data.

The prognostic value of the miRNA signature for OS of patients in the early stage of disease or with different smoking status was also assessed using the survival ROC analysis. We also validated the prognostic utility of the linear miRNA prognostic model in TCGA lung SCC patients.

To evaluate the contribution of miRNAs as independent prognostic factors of patient survival, we used a multivariate analysis. All variables reaching a significant level of 10% in univariate analyses were tested in a Cox proportional hazards model. All reported *P* values were two-sided. All analyses were performed using the R/BioConductor (version 3.0.2) [19] and survival curves and ROCs were generated by ggplot2, survMisc and survivalROC [20] packages.

### In *silico* analysis of pathways specifically targeted by the prognostic miRNAs in LUAD

We examined whether altered miRNA expression associated with OS had a functional effect on the progression of LUAD. The miRWalk online database [21], which offers a comparative platform of possible miRNA-target predictions using 10 different data sets in addition to validated targets, was used to predict target genes of the eight miRNAs. The target gene was selected if it was predicted by at least three data sets using miRanda, miRDB, miRWalk, PITA, RNA22 and Targetscan programs. Over-representation analysis (ORA) was performed using the GeneTrail gene set analysis tool [22,23] with default settings to detect the potential biological terms or functional effect categories represented in the target gene list. The *P* values for the biological categories were adjusted by FDR and were considered statistically significant at *P* < 0.05.

## Results

### Identification of differentially expressed miRNAs in LUAD patients

Analysis of miRNA expression profiles in LUAD patient tissues (n = 385) compared with normal lung tissues (n = 46) identified a total of 111 differentially expressed miRNAs (logFC > 1 or logFC < -1, *P* < 0.05 after FDR adjustment), which were used for subsequent survival analyses (Additional file 1: Table S1). Of these, 82 miRNAs were over-expressed including miR-31 and miR-196b, which exhibited > 8-fold increased expression. 29 miRNAs were down-regulated, including miR-187, miR-331 and miR-101-1.

### Correlation between miRNA expression, clinical features and prognosis in the TCGA LUAD cohort

Clinical covariates for LUAD patients are summarized in Table 1. Owing to the high censoring rate (69.35%) in the TCGA LUAD cohort, which refers to patients who may leave the study or are still alive at the end of the study, we first performed univariate survival analyses. This was used to confirm the prognostic significance of previously established clinical parameters in the cohort, including stage, age and other clinicopathological features.

**Table 1:** Clinical covariates for the TCGA lung adenocarcinoma cohort in the training and test set

**Covariates**		**Total**	**Training set**	**Testing set**	***P*-value**
		**N = 372**	**N = 186**	**N = 186**	
Age, years, no (%)	<=65	174 (46.8)	94 (50.5)	80 (43.0)	0.1767
	>65	198 (53.2)	92 (49.5)	106 (57.0)	
Gender, no (%)	Male	176 (47.3)	87 (46.8)	89 (47.8)	0.9173
	Female	196 (52.7)	99 (53.2)	97 (52.2)	
Vital status	Alive	258 (69.4)	125 (67.2)	133 (71.5)	0.4311
	Dead	114 (30.6)	61 (32.8)	53 (28.5)	
Disease stage, no (%)	I	199 (53.5)	100 (53.8)	99 (53.2)	0.9000
	II	89 (23.9)	42 (22.6)	47 (25.3)	
	III	66 (17.7)	35 (18.8)	31 (16.7)	
	IV	17 (4.6)	9 (4.8)	8 (4.3)	
Lymph node	N0	231 (62.1)	118 (63.4)	113 (60.8)	0.6046
Involvement, no (%)	N1	72 (19.4)	32 (17.2)	40 (21.5)	
	N2	58 (15.6)	32 (17.2)	26 (14.0)	
	N3	1 (0.3)	0 (0.0)	1 (0.5)	
M stage, no (%)	M0	266 (71.5)	131 (70.4)	135 (72.6)	0.4400
	M1	16 (4.3)	10 (5.4)	6 (3.2)	
T stage, no (%)	T1	112 (30.1)	52 (28.0)	60 (32.3)	0.6320
	T2	211 (56.7)	108 (58.1)	103 (55.4)	
	T3	31 (8.3)	15 (8.1)	16 (8.6)	
	T4	16 (8.3)	10 (5.4)	6 (3.2)	
Smoking status	Nonsmoker	143 (38.4)	66 (35.5)	77 (41.4)	0.3214
	Smoker	217 (58.3)	113 (60.8)	104 (55.9)	
Adjuvant treatment	None	255 (68.5)	121 (65.1)	134 (72.0)	0.5530
	Chemotherapy	68 (18.3)	37 (19.9)	31 (16.7)	
	Radiotherapy	20 (5.4)	13 (7.0)	7 (3.8)	
	Chemoradiotherapy	27 (7.3)	14 (7.5)	13 (7.0)	
	Other	2 (0.5)	1 (0.5)	1 (0.5)	

Nonsmoker: lifetime nonsmoker or current reformed smoker for > 15 years.

**Figure 1:**
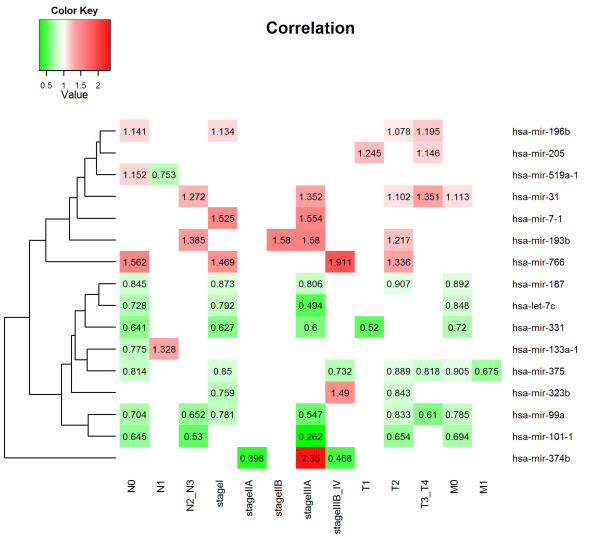
**MiRNAs associated with prognosis in different clinical subclasses of TCGA lung adenocarcinoma cohort.** The matrix visualizes the significant HRs for the 16 miRNAs in the TCGA LUAD cohort. Numbers in the rectangles indicate the HRs for expression with significant univariate Cox regression (P < 0.1). Red rectangles indicate HRs >1 and blue rectangles indicate HRs <1.

Clinical variables of N stage, T stage, M stage and disease stage were significantly associated with OS; however, age, gender, smoking status and adjuvant treatment were not. Kaplan-Meier survival curves for these variables are shown in the Additional file 1: Figures S1–S8. The results of this preliminary assessment indicated that despite the high level of censored data in this cohort, the survival data for the TCGA LUAD cohort were informative and suitable for studying the prognostic relevance of miRNA expression.We next conducted univariate survival analyses to identify common miRNAs related to OS within each of the following independent classes: disease stage, N stage, M stage and T stage. Within each subset of clinical characteristics, the patient subclasses represented non-overlapping sets. MiRNAs associated with OS, exhibiting a significance level of 10% in at least two independent categories for each covariate, were selected as candidate markers. The respective HRs for the common miRNA expression in each subclass are shown in Figure 1.

Eight miRNAs were selected, based on the importance scores computed by the supervised principal component method in the training set. A mathematical formula with eight miRNAs was then constructed for clinical outcome prediction. The same prognostic score formula obtained from the training set was used to calculate the eight-miRNA signature score for each of the 186 patients in the testing set.

**Figure 2:**
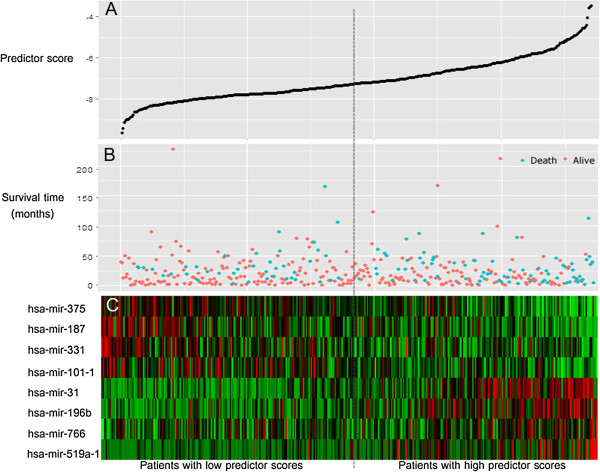
**MicroRNA predictor-score analysis of 372 LUAD patients in TCGA cohort. (A)** MicroRNA predictor-score distribution. **(B)** Patients’ survival status. **(C)** Color-gram of miRNA expression profiles of LUAD patients. The blue dotted line represents the median miRNA signature cutoff dividing patients into low-risk and high-risk groups.

Figure 2 shows the distribution of patient prognostic scores, the survival status and tumor miRNA expression of all 372 LUAD patients, ranked according to the prognostic score values for the eight-miRNA signature. Of these eight miRNAs, four were associated with high risk (hsa-mir-31, miR-196b, miR-766, miR-519a-1, HR > 1) and four were shown to be protective (miR-375, miR-187, miR-331, miR-101-1, HR < 1). Tumors with high prognostic scores tended to express high-risk miRNAs, whereas tumors with low prognostic scores tended to express protective miRNAs (Figure 2B and C). Patients with high-risk scores had more deaths than low-risk-score patients. Similar results were observed in both the training set and the testing set. We also compared the expression of the eight-miRNA signature between short-term (fatal within 2 years, n = 138, those who were censored within 2 years not included) and long-term survivors (n = 57). The eight-miRNA signature scores between long- and short-term survivors were significantly different (*P* = 0.0011). Recurrence (local or regional, or distant) data were available for 263 cases in TCGA LUAD cohort. High miRNAs signature score was also related to short recurrence free survival (HR = 1.262, *P* = 0.011) in this subset.

**Figure 3:**
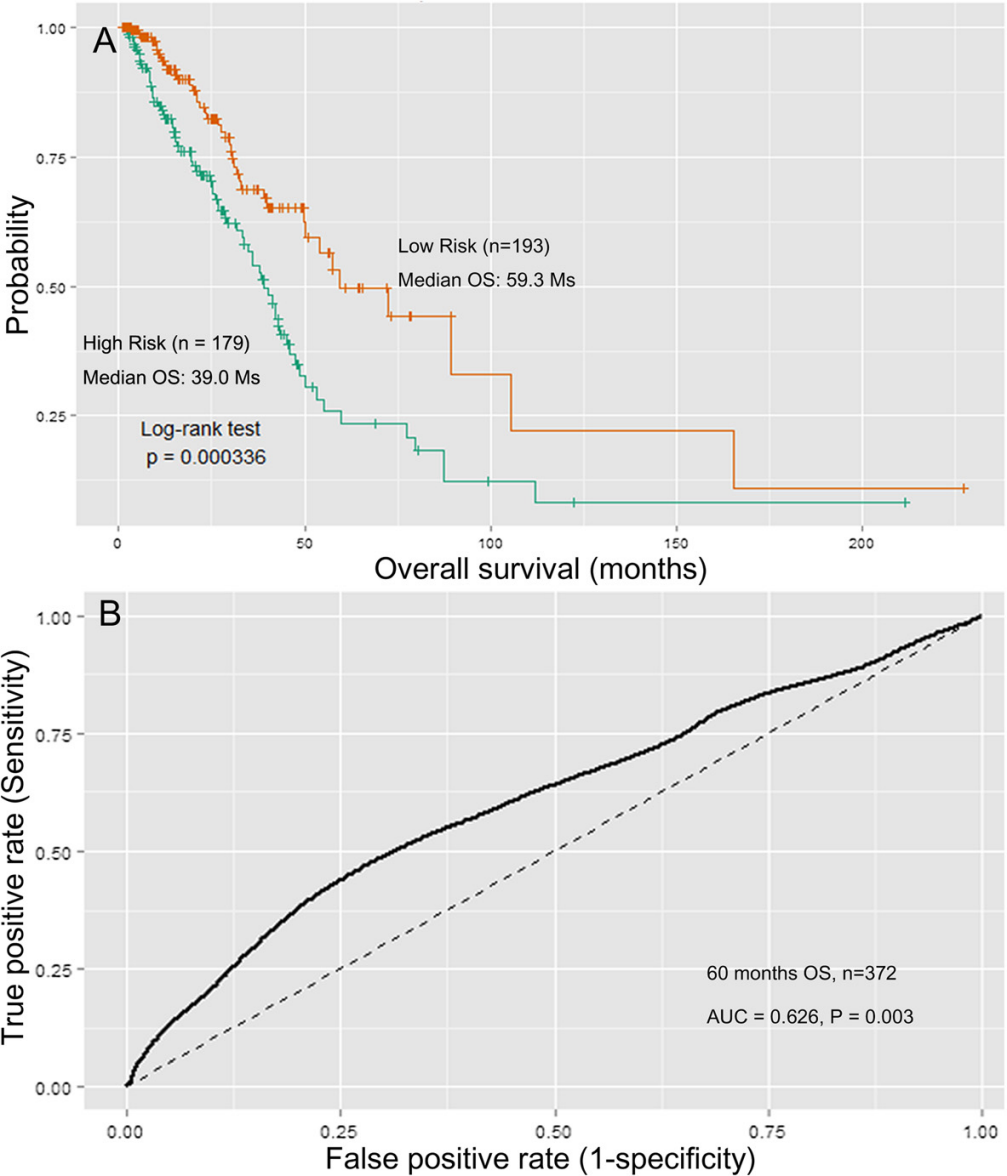
**Kaplan–Meier and ROC curves for the 8-miRNA signature in TCGA LUAD cohort. (A)** The Kaplan–Meier curves for LUAD risk groups obtained from the TCGA cohort (n = 372) divided by the median cutoff point. Patients with high scores had poor outcome in terms of OS (Median OS: 39.0 months *vs.* 59.3 months, *P* < 0.001). **(B)** The ROC curve had an AUC of 0.626 (*P* = 0.003). The permutation *P* value was obtained from 1,000 permutations for testing the null hypothesis (AUC = 0.5).

The median cutoff point obtained from the training set was used for the entire TCGA LUAD patient cohort to classify the patients into either high-risk or low-risk groups. Comparison of clinicopathological factors in the high- and low-risk groups (Additional file 1: Table S2) revealed that the eight-miRNA signature was significantly correlated with lymph node metastasis (*P* = 0.0085) and clinical stage (*P* = 0.0252). Patients expressing the high-risk miRNA signature exhibited poorer OS than patients expressing the low-risk miRNA signature (median OS of 39.0 months *vs.* 59.3 months, HR = 1.99, *P* value < 0.001). Kaplan-Meier curves for the high-risk and low-risk groups within the TCGA LUAD cohort (n = 372) are shown in Figure 3A. Time-dependent ROC curves were used to assess the prognostic power of the eight-miRNA signature. The AUC for the eight-miRNA signature prognostic model was 0.626 at 60 months of OS (*P* = 0.003, Figure 3B). However, eight-miRNA signature was not significantly associated with OS of lung SCC patients (HR = 1.200, *P* = 0.380) and the AUC was 0.522 (*P* = 0.397).

### Independent prognostic value of miRNA signatures

Since patients at the early tumor stage may benefit significantly from a prognostic biomarker signature, we also evaluated the prognostic power of the eight-miRNA signature in stage I and II LUAD tumors (n = 288). This signature also demonstrated good performance on early tumors (AUC = 0.605, permutation *P* = 0.027, Additional file 1: Figure S9).

**Figure 4:**
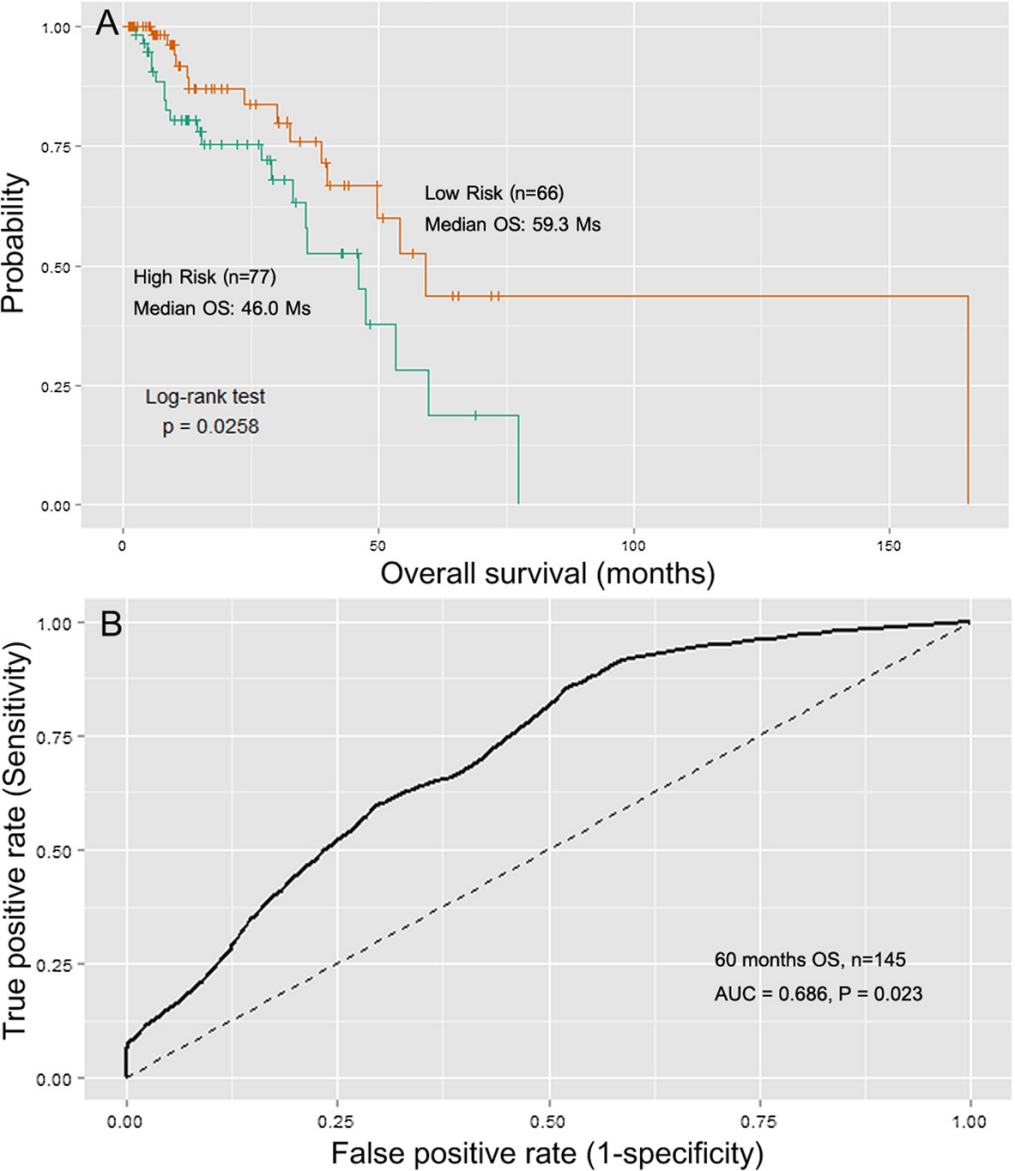
**Kaplan–Meier and ROC curves for the 8-miRNA signature in TCGA non-smoking LUAD cohort. (A)** The Kaplan–Meier curves for LUAD risk groups obtained from the TCGA non-smoking LUAD cohort (n = 145) divided by the median cutoff point. Non-smoking patients with high scores had poor outcome in terms of OS (Median OS: 46.0 months *vs.* 59.3months, *P* = 0.027). **(B)** The ROC curve had an AUC of 0.686 (P = 0.023). The permutation P value was obtained from 1,000 permutations for testing the null hypothesis (AUC = 0.5).

Surprisingly, although there was no relationship between smoking status and OS in the TCGA LUAD cohort, the eight-miRNA signature exhibited superior prognostic value for patients who were non-smoking or reformed smokers for more than 15 years (n = 145). The AUC at 60 months for this subgroup was 0.686 with a permutation *P* value of 0.023 (Figure 4). Further analysis indicated that smoking status was significantly related to age (*P* = 0.000, Additional file 1: Table S3); however, the AUC for the younger population (diagnosed before 65 year-old) was not better than that for the entire cohort (AUC = 0.593, *P* < 0.05).

We also conducted a multivariate analysis to evaluate the independent prognostic value of the eight-miRNA signature. All variables reaching a significant level of 10% in univariate analyses were tested in a Cox proportional hazards model. The miRNA signature, T stage, N stage and M stage were used as covariates and age was also included into the multivariate model as a potential confounding risk factor. Tumor stage was not included owing to its interaction with TNM staging system. This analysis revealed that the miRNA signature (HR = 1.493, *P* < 0.001) and the lymph node involvement (N stage) (HR = 2.607, *P* < 0.001) are independent prognostic factors associated with OS (Additional file 1: Table S4).

### In *silico* analysis of pathways specifically targeted by the prognostic miRNAs in LUAD

We used GeneTrail to identify functional categories among target genes that could be predicted by the selected miRNAs. A total of 7686 target genes were identified as potentially regulated by miRNAs contained in the eight-miRNA signature. We performed an ORA to test the specific functional categories of genes from Kyoto Encylopedia of Genes and Genomes (KEGG) categories and 852 Gene Ontology (GO) categories that are targeted by miRNAs. It revealed enrichment of 55 KEGG categories and 852 GOcategories (*P*-values < 0.05 after FDR adjustment, Additional file 1: Table S5). This analysis revealed an overrepresentation of the predicted miRNA targets involved in the critical pathway linked to tumor-promoting function such as: focal adhesion, adherens junction, apoptosis, Ras protein signal transduction, and p53 signaling pathway We observed an overlap of target genes enriched in cancer-related KEGG categories of NSCLC and small cell lung cancer (SCLC). These indicate a potentially important functional role of selected miRNAs in the progression of lung cancer. The experimentally validated target genes (obtained from miRWalk) involved in the pathways related to tumor-promoting function with highly significant *P*-values were shown in Table 2. Taken together, these exploratory analyses suggest that variation in miRNAs expression might affect the critical pathways involved in LUAD progression, an important mechanism warranting follow-up research.

**Table 2:** Results of the over-representation analysis of the predicted target genes

**Subcategory name**	**P-value (FDR)**	**Expected**	**Observed**	**Involved experimentally validated target genes of the prognostic miRNA**	
NSCLC	0.00336054	21.2903	34	miR-31	CCND1 CDKN2A E2F2 E2F3 KRAS P53
				miR-196b	CCND1
				miR-187	KRAS
				miR-519a-1	CCND1
				miR-375	AKT2 CDK6 PDPK1
				miR-101-1	CDKN2A PRKCA
SCLC	0.00918204	33.1182	47	miR-31	CCND1 CDKN2B TP53
				miR-196b	BCL2 CCND1
				miR-519a-1	BCL2
				miR-375	AKT2 CDK6
				miR-101-1	ITGA2 ITGA3 ITGAV
Apoptosis	0.00558373	34.6953	50	miR-31	MYD88 TNF TP53
				miR-196b	BCL2
				miR-519a-1	ATM BCL2
				miR-375	AKT2
				miR-101-1	ATM
p53 signaling pathway	0.0149892	27.2043	39	miR-31	CCND1 TP53
				miR-196b	CCND1
				miR-519a-1	CCND1 GADD45A
				miR-375	CDK6
				miR-101-1	ATM CDKN2A
Ras protein signal transduction	2.20423e-07	87.7829	131	miR-31	CDKN2A FGF2 KRAS TIAM1 TP53
				miR-187	KRAS
				miR-375	MAPK3 MAPK14
				miR-101-1	BCR CDKN2A CSF1

## Discussion

In this study, we identified 16 miRNAs correlated with OS of LUAD patients in different clinical classes, from the 111 most significantly altered miRNAs in LUAD tissues compared with normal lung tissues. A linear combination of eight miRNAs (miR-31, miR-196b, miR-766, miR-519a-1, miR-375, miR-187, miR-331 and miR-101-1) was validated as an independent predictor for LUAD patient survival. This signature demonstrated significant prognostic performance in both the entire LUAD cohort and the early stage subgroup, particularly in the non-smoking or reformed smoker (more than 15 years) group. Our results suggest that there is a potential role for miRNAs in the molecular pathogenesis, clinical progression and prognosis of LUAD, and highlights the potential of miRNA profiling to improve clinical prognosis in patients with LUAD.

LUAD, constitutes about 30 - 40% of NSCLC, and is a global public health problem, representing the most common cause of cancer-related death [1]. Owing to immense heterogeneity from multiple aspects (pathology, molecular, clinical, radiology and surgery)observed in LUAD patients, the development of individualized cancer treatment and prediction of patient outcome have been huge challenges [24]. In the past decade, several molecular markers and models have been proposed or developed within specific NSCLC subgroups. In particular, the identification of driver mutations in the EGFR and anaplastic lymphoma kinase (ALK), introduced a new era of targeted therapy in LUAD [25,26]. Treatment choice and monitoring of patient outcome based on the analysis of mutations in other key biomarkers including *Her2, PIK3CA, BRAF, NUTM1, MET, ROS1, FGFR1*, *KRAS* and *PTEN* may also have a potentially powerful clinical impact [27-29]. Furthermore, gene expression profiling by microarrays or RT-PCR has also been used to classify or predict prognosis in patients with lung cancer. Owing to the large numbers of genes and the low prevalence of mutations, it may be more effective to use miRNA rather than gene expression profiles, to classify various cancer subtypes [30]. MiRNAs are small, conserved non-coding regulatory RNAs in humans, and they play important roles in carcinogenesis. Each miRNA may post-transcriptionally regulate hundreds of downstream genes by targeting the 3’ untranslated region of specific messenger RNAs for degradation or translational repression [5,31]. While still in the early stages of clinical development, miRNA-expression profiling of primary tumors has already demonstrated significant promise in clinical stratification and monitoring of therapy [32].

Several groups have identified miRNA signatures capable of predicting clinical outcome in NSCLC patients. In one miRNA profiling study based on a cohort of 357 stage I NSCLC patients, a miRNA expression signature containing 27 miRNAs was identified that was capable of accurately predicting which stage I LUAD patients may benefit from more aggressive therapy [10]. A study of 112 NSCLC patients (57 squamous cell carcinoma [lung SCC] and 60 LUAD, stage I- III, Asian patients) identified a five-miRNA signature (including miR-221, let-7a, miR-137, miR-372 and miR-182∗) as an independent predictor of cancer relapse and survival [7]. Another study, screening serum miRNAs using Solexa sequencing, followed by a self-validated study of 303 patients, identified miR-486, miR-30d, miR-1 and miR-499 as non-invasive predictors of OS in NSCLC [6]. Boeri *et al.* also found that higher levels of miR-429 correlated with a worse disease-free survival in lung cancer [33]. A recent study confirmed three novel miRNAs (miR-662, miR -192 and miR -192*) as prognostic for distant relapse in operable lung SCC [34]. In addition, miR-708 was shown to be associated with poor survival in LUAD from patients who had never smoked [11]. On the basis of these studies, miRNA profiling has already demonstrated significant potential as a prognostic indicator in lung cancer. However, it should be noted that there was little overlap between the miRNAs identified as prognostic predictors of disease progression or outcome in these various studies, indicating that comprehensive validation of miRNAs identified in these screens is necessary.

These inconsistencies may be caused, at least in part, by fundamental, methodological differences in the pre-selection of candidate miRNAs. In this study, TMM normalization and the GLM method (which accounts for the sampling properties of RNA-seq data and the batch effect, respectively) were used to obtain differentially expressed miRNAs between tumor and normal tissues. Moreover, we obtained the candidate miRNAs from a list of differentially expressed miRNAs between LUAD and normal samples. This method ensured that the prognostic microRNA signature had statistically altered expression in LUAD and also had a prognostic impact on survival. However, miRNAs associated with OS and those related with occurrence of LUAD may not completely overlap. It is another reason for the discrepancy in miRNAs identified between various studies. The discrepancy may also be due to differences in sample size, individual patients or the study population or the different platforms used. Since miRNA expression profiles strongly differ between LUAD and lung SCC [8], the LUAD-specific target miRNAs identified in this study may have further potential application in predicting the clinical outcome in patients with LUAD and revealing targets for the development of therapy.

In this study, we selected only common miRNAs related to clinical outcome in the non-overlapping subclasses, from the same class as the potential prognostic miRNAs. For this reason, several of the miRNAs previously identified as being associated with OS in lung cancer were not obtained, since they were only significant within a single subclass in the TCGA cohort. Among the eight miRNAs, miR-31 has been validated as a marker for lymph node metastasis in lung cancer [35]. MiR-31 has been shown to act as an oncogenic miRNA by targeting specific tumor suppressors, including the large tumor suppressor 2 (LATS2) and PP2A regulatory subunit B alpha isoform (PPP2R2A) [36], its high expression has been associated with poor survival of lung SCC [37]. In contrast, in a study of 164 NSCLC patients, low miR-375 expression in plasma was associated with worse OS [9]. Down regulation of miR-375 in tissues was also significantly associated with poor outcome in patients with esophageal SCC [38]. It was proved that miR-101 expression was significantly associated with pathological stage and lymph node involvement, and might play an important role as a biomarker for prognosis and therapeutic targets of NSCLC [39], (through directly targeting enhancer of zeste homolog 2(EZH2) [40]). For the remaining five miRNAs, to our knowledge, there are no associations reported between these and OS in lung cancer. MiR-196b has been identified as a biomarker, capable of distinguishing lung SCC and LUAD [41]. It also demonstrates potential prognostic value for disease progression in gastric cancer and glioblastoma [42,43]. Although there was no obvious evidence of an association between miR-196b and OS in lung cancer, Annexin A1, one of several validated miR-196b target genes, has been identified as a pro-invasive and prognostic factor for in LUAD [44]. Ectopic expression of miR-187 was reported to lead to a significantly more aggressive phenotype in breast cancer cells and clear cell renal cell carcinoma [45,46]. Deregulation of miR-519a-1, regulated by phospho (p)-ΔNp63α, in head and neck SCC cells, led to the subsequent modulation of several target mRNAs including TP73, YES1, PARP1, HIPK2, ATM, CDKN1A, CASP3, DDIT4, BCL2 and BCL2L2, and YAP1, that are involved in apoptotic processes [47]. Similarly, overexpression of miR-766 was shown to significantly inhibit the expression of pro-apoptotic genes caspase-3 and Bax in acute promyelocytic leukemia cells [48]. Previous studies have also shown that miR-331-3p, a member of miR-331 family, may be involved in cell cycle control by targeting the 3′-untranslated region of the cell cycle-related molecule, E2F1 [49]. The ORA in this study also revealed a significant enrichment of miRNA targets involved in NSCLC and SCLC KEGG pathways. Genes involved in apoptosis/regulation of cell cycle, the categories which were enriched within the target genes of our eight miRNAs, are implicated in LUAD tumorigenesis and represent potential therapeutic targets [50]. Several genes involved in these pathways, such as AKT2, TP53 and TNF, have been identified as the key biomarker of LUAD prognosis [51-53]. Our in silico pathway enrichment analysis based on the predicted target mRNA genes, suggested that variation in miRNAs expression might affect critical pathways involved in LUAD progression. Since all target prediction algorithms generate certain fraction of both false positives and false negatives, further research is warranted.

Lung cancer in non-smokers has recently been recognized as a distinct disease entity, owing to the striking demographic, clinicopathological and molecular differences between lung cancer in never-smokers and ever-smokers [54,55]. Due to its prominence in Asian countries and increasing trend in most developed countries [56], investigations and clinical trials should be undertaken to determine the underlying causes and factors affecting progression of non-smoking-related lung cancer.

Several studies have linked smoking to poor outcomes among patients with lung cancer [57-59]. However in TCGA LUAD cohort, there was no significant difference in OS between smoking and non-smoking groups (median survival time: 42.9 months *vs.* 49.7 months). Intriguingly, we found that the eight-miRNA signature exhibited superior performance in predicting the 5-year survival of patients with lung cancer who had never smoked or who had ceased smoking more than 15 years ago. To examine the difference in AUCs, we compared the clinical characteristics between smoking and non-smoking groups. We found the only significant correlation between smoking history and clinicopathological features to be age. Smoking is more common among young patients in TCGA LUAD cohort. About 72.4 per cent (126 of 174) of TCGA LUAD patients diagnosed at a young age (<or = 65 years), were current smokers or reformed smokers of less than 15 years. However, there was no significant association of young age with poor OS and we did not find-better AUC in young age groups. This suggests that miRNA profile of the smoking- and non-smoking-related lung cancer may be fundamentally different, requiring further study. Previous reports have shown that some of the eight miRNAs identified in this study, such as miR-31 and miR-101, to be potential cigarette smoke-mediated deregulated miRNAs in lung cancer [60]. This prognostic miRNA signature classifier for non-smoking-related LUAD may help clinicians to pinpoint those LUAD patients at high risk of unfavorable OS.

There are number of limitations to this study. A major limitation was the lack of available information regarding adjuvant therapy and EGFR mutation status, which defines distinct molecular subsets of resected LUAD and also predicts whether tumors are sensitive to EGFR tyrosine kinase inhibitors [53]. Such information is required to further study the interaction between the prognostic effect of their status and the miRNA signature. A further limitation was that the TCGA LUAD cohort had a relatively short follow-up period (median follow-up of 15 months) and the censored rate was high, which may affect the reliability of the Kaplan-Meier estimates. There are also limitations in obtaining all the data from a single source and randomly assigning samples to training and testing sets for the development and assessment of the prognostic model. Independent external validation sets with long-term follow up to provide a realistic assessment of the performance of this miRNA signature would be more reliable.

## Conclusions

We have identified a miRNA signature comprising eight miRNAs (miR-31, miR-196b, miR-766, miR-519a-1, miR-375, miR-187, miR-331 and miR-101-1), which can be used as an independent prognostic marker of LUAD patient survival. The independent prognostic model demonstrated good performance in predicting 5-year survival, especially in non-smokers. This signature may help to identify LUAD patients at high risk of recurrence or metastasis, who may benefit from adjuvant therapy. However, a number of limitations to this study exist. The major limitation involves the lack of available information regarding adjuvant therapy. Such information is required to further study the interaction between this miRNA signature and adjuvant therapy. An independent validation of this miRNA signature is also required.

## Abbreviations

ALK: Anaplastic lymphoma kinase; AUC: Area under the respective ROC curves; EGFR: Epidermal growth factor receptor; FDR: False discovery rate; GLM: Generalized linear model; GO: Gene ontology; HR: Hazard ratio; KEGG: Kyoto encylopedia of genes and genomes; logFC: Log 2 fold change; LUAD: Lung adenocarcinoma; miRNA: miR, microRNA; NSCLC: Non-small cell lung cancer; ORA: Over-representation analysis; OS: Overall survival; ROC: Receiver operating characteristic; TCGA: The Cancer genome Atlas; TMM: Trimmed mean Of M values method; SCLC: Small cell lung cancer.

## Competing interests

The authors declare that they have no of interest.

## Authors’ contributions

XLL designed the study, performed data analysis and drafted the manuscript. YRS participated in the collection and analysis of data. ZHY and XXX verified the bioinformatics analysis. BSZ conceived the study and participated in its design and coordination. All authors read and approved the final manuscript.

## Supplementary Material

Additional file 1: Table S1 The differentially expressed miRNAs in LUAD and normal samples. **Table S2.** Clinical covariates for the TCGA LUAD cohort by miRNA signature group. **Table S3.** Clinical covariates for the TCGA LUAD cohort by smoking status. **Table S4.** Multivariate Cox proportional hazards analysis. **Table S5.** The over-representation analysis for target genes. **Figure S1.** The prognostic role of disease stage in LUAD. **Figure S2.** The prognostic role of N stage in LUAD. **Figure S3.** The prognostic role of M stage in LUAD. **Figure S4.** The prognostic role of T stage in LUAD. **Figure S5.** The prognostic role of therapy in LUAD. **Figure S6.** The prognostic role of age in LUAD. **Figure S7.** The prognostic role of gender in LUAD. **Figure S8.** The prognostic role of smoking status in LUAD. **Figure S9.** Kaplan–Meier and ROC curves for the 8-miRNA signature for patients of stage I and II. **(A)** The Kaplan–Meier curves for LUAD risk groups o for patients of stage I and II (n = 288) divided by the median cutoff point. **(B)** The ROC curve had an AUC of 0.605 (P = 0.027). Click here for file
