# Supplementary material for: An eight-miRNA signature as a potential biomarker for predicting survival in lung adenocarcinoma
Source: J Transl Med. 2014 Jun 4;12:159. doi: 10.1186/1479-5876-12-159 (PMC4062505; doi:10.1186/1479-5876-12-159)
Supplement: Additional file 1: Table S1 — The differentially expressed miRNAs in LUAD and normal samples. Table S2. Clinical covariates for the TCGA LUAD cohort by miRNA signature group. Table S3. Clinical covariates for the TCGA LUAD cohort by smoking status. Table S4. Multivariate Cox proportional hazards analysis. Table S5. The over-representation analysis for target genes. Figure S1. The prognostic role of disease stage in LUAD. Figure S2. The prognostic role of N stage in LUAD. Figure S3. The prognostic role of M stage in LUAD. Figure S4. The prognostic role of T stage in LUAD. Figure S5. The prognostic role of therapy in LUAD. Figure S6. The prognostic role of age in LUAD. Figure S7. The prognostic role of gender in LUAD. Figure S8. The prognostic role of smoking status in LUAD. Figure S9. Kaplan–Meier and ROC curves for the 8-miRNA signature for patients of stage I and II. (A) The Kaplan–Meier curves for LUAD risk groups o for patients of stage I and II (n = 288) divided by the median cutoff point. (B) The ROC curve had an AUC of 0.605 (P = 0.027). [file 1479-5876-12-159-S1.pdf]

Table S1. The differentially expressed miRNAs in LUAD and normal samples

miRNA	logFC	logCPM	LR	*P*-value	FDR
hsa-let-7a-1	-1.17679	13.33789	148.6665	3.39E-34	5.39E-33
hsa-let-7a-2	-1.17534	14.34003	149.2303	2.55E-34	4.31E-33
hsa-let-7a-3	-1.17449	13.34533	148.3797	3.92E-34	5.88E-33
hsa-let-7c	-1.57564	10.69916	122.141	2.15E-28	2.42E-27
hsa-let-7f-2	-1.4696	13.87424	188.7286	6.02E-43	1.36E-41
hsa-mir-1-2	-2.59898	4.065747	133.8718	5.83E-31	7.87E-30
hsa-mir-101-1	-1.27797	13.66537	135.6156	2.42E-31	3.44E-30
hsa-mir-101-2	-2.13637	5.814561	321.1529	8.12E-72	2.19E-69
hsa-mir-105-1	6.383098	4.822748	30.87248	2.76E-08	8.00E-08
hsa-mir-105-2	7.427085	4.822094	36.99548	1.18E-09	4.15E-09
hsa-mir-1247	-1.66999	4.743856	39.15314	3.92E-10	1.43E-09
hsa-mir-126	-1.50625	11.34904	161.1408	6.37E-37	1.15E-35
hsa-mir-1266	1.18216	4.261688	30.15171	4.00E-08	1.14E-07
hsa-mir-1269	8.979179	7.09475	77.94129	1.06E-18	7.54E-18
hsa-mir-129-1	1.397652	2.305911	13.50877	0.000237	0.000445
hsa-mir-129-2	1.475944	2.46905	15.1715	9.82E-05	0.000198
hsa-mir-1301	1.559138	3.930829	65.23428	6.65E-16	3.74E-15
hsa-mir-1306	1.265995	3.350879	43.3168	4.66E-11	1.82E-10
hsa-mir-1307	1.46029	10.43267	108.2041	2.42E-25	2.42E-24
hsa-mir-130b	2.013942	5.11708	92.51654	6.68E-22	5.46E-21
hsa-mir-133a-1	-2.34487	3.150875	107.4977	3.46E-25	3.34E-24
hsa-mir-134	1.386782	7.816498	24.38979	7.87E-07	2.00E-06
hsa-mir-135b	2.256789	5.941297	70.39904	4.84E-17	2.84E-16
hsa-mir-140	-1.24746	10.08169	179.3729	6.64E-41	1.38E-39
hsa-mir-141	1.427523	10.57744	70.50162	4.60E-17	2.76E-16
hsa-mir-143	-1.94447	17.04544	299.298	4.68E-67	5.38E-65
hsa-mir-144	-3.39005	7.817757	239.4497	5.18E-54	2.00E-52
hsa-mir-149	1.12099	5.195308	15.70989	7.38E-05	0.000151
hsa-mir-153-2	3.340968	4.170767	86.81247	1.19E-20	8.95E-20
hsa-mir-154	1.078276	2.638742	13.62325	0.000223	0.000422
hsa-mir-182	1.886096	14.46114	105.3396	1.03E-24	9.26E-24
hsa-mir-183	2.346508	13.47908	115.4896	6.15E-27	6.64E-26
hsa-mir-184	-3.55362	3.533951	132.5622	1.13E-30	1.45E-29
hsa-mir-187	2.235523	4.892498	31.58536	1.91E-08	5.73E-08
hsa-mir-192	2.851677	11.19809	36.87095	1.26E-09	4.31E-09
hsa-mir-193b	2.128594	6.168042	77.86821	1.10E-18	7.63E-18
hsa-mir-194-1	2.715447	8.867929	35.02366	3.26E-09	1.07E-08
hsa-mir-194-2	2.690178	8.993801	34.50296	4.26E-09	1.37E-08
hsa-mir-195	-2.06901	5.739235	298.812	5.98E-67	5.38E-65
hsa-mir-196a-1	6.09268	6.847917	75.35353	3.94E-18	2.59E-17
hsa-mir-196a-2	5.159921	2.438671	45.43333	1.58E-11	6.66E-11

hsa-mir-196b	3.069759	6.783817	43.63745	3.95E-11	1.57E-10
hsa-mir-200a	2.025154	9.799207	92.15972	8.00E-22	6.35E-21
hsa-mir-200b	1.048135	9.544366	30.46155	3.41E-08	9.78E-08
hsa-mir-204	-1.04731	2.623073	11.75746	0.000606	0.001069
hsa-mir-205	2.954848	9.007248	31.27737	2.24E-08	6.64E-08
hsa-mir-21	1.668484	18.40611	169.6405	8.86E-39	1.71E-37
hsa-mir-210	4.536205	9.981875	235.0115	4.81E-53	1.62E-51
hsa-mir-215	3.303523	5.506995	36.96169	1.20E-09	4.17E-09
hsa-mir-217	1.28307	5.3757	17.20751	3.35E-05	7.24E-05
hsa-mir-218-2	-2.1717	5.94516	282.303	2.36E-63	1.60E-61
hsa-mir-223	-1.5356	8.391868	77.42393	1.38E-18	9.31E-18
hsa-mir-224	1.599938	5.823381	28.68716	8.51E-08	2.34E-07
hsa-mir-296	1.084033	2.647393	17.8237	2.42E-05	5.28E-05
hsa-mir-30a	-2.46106	14.54987	233.2685	1.15E-52	3.46E-51
hsa-mir-30c-2	-1.35665	8.912388	203.4176	3.75E-46	9.21E-45
hsa-mir-31	3.380597	4.229678	40.77186	1.71E-10	6.42E-10
hsa-mir-323	2.547688	2.52574	28.18781	1.10E-07	3.00E-07
hsa-mir-323b	4.15596	3.022184	53.79887	2.22E-13	1.07E-12
hsa-mir-331	1.410294	5.494887	105.4691	9.64E-25	8.97E-24
hsa-mir-338	-1.21074	9.663072	28.73095	8.32E-08	2.31E-07
hsa-mir-33a	1.036796	4.395712	32.88705	9.77E-09	3.03E-08
hsa-mir-345	2.041154	4.208743	90.27147	2.08E-21	1.60E-20
hsa-mir-34b	-1.54076	4.333465	18.0358	2.17E-05	4.80E-05
hsa-mir-34c	-1.99601	6.731809	33.32002	7.82E-09	2.48E-08
hsa-mir-3607	2.214981	5.289925	126.5558	2.32E-29	2.85E-28
hsa-mir-370	1.181339	3.271463	14.24898	0.00016	0.000311
hsa-mir-371	4.46682	3.243685	7.820083	0.005167	0.007972
hsa-mir-372	4.767824	6.167704	15.13525	0.0001	0.0002
hsa-mir-373	4.210204	1.868783	9.930946	0.001625	0.002683
hsa-mir-374b	-1.36069	5.928331	230.5466	4.53E-52	1.22E-50
hsa-mir-375	2.075957	15.18177	36.50564	1.52E-09	5.14E-09
hsa-mir-378	-1.24228	8.408202	32.88942	9.76E-09	3.03E-08
hsa-mir-381	1.032088	5.596999	14.25305	0.00016	0.000311
hsa-mir-382	1.33107	4.065253	23.36398	1.34E-06	3.35E-06
hsa-mir-409	1.929186	4.577288	39.87918	2.70E-10	9.99E-10
hsa-mir-410	1.600596	4.27548	23.27381	1.40E-06	3.48E-06
hsa-mir-412	1.352283	2.173683	12.62103	0.000381	0.000696
hsa-mir-424	1.189727	6.395309	36.40273	1.60E-09	5.35E-09
hsa-mir-429	1.049187	6.926867	27.54045	1.54E-07	4.15E-07
hsa-mir-431	1.98523	4.087955	25.58623	4.23E-07	1.11E-06
hsa-mir-432	1.074869	3.295471	10.11221	0.001473	0.00247
hsa-mir-451	-3.39584	9.901368	247.7446	8.06E-56	3.63E-54
hsa-mir-455	1.20721	7.384144	64.8525	8.07E-16	4.45E-15

hsa-mir-483	1.608098	3.130996	9.824578	0.001722	0.002818
hsa-mir-485	1.712744	2.869282	20.10235	7.34E-06	1.72E-05
hsa-mir-486	-3.27573	8.53928	248.0266	6.99E-56	3.63E-54
hsa-mir-487b	1.336885	2.747046	16.39786	5.13E-05	0.000107
hsa-mir-493	1.291799	3.751886	20.94234	4.73E-06	1.12E-05
hsa-mir-509-2	1.173383	2.639822	5.065098	0.024412	0.034691
hsa-mir-519a-1	2.278719	2.984921	7.959426	0.004784	0.007466
hsa-mir-539	2.768769	2.756358	44.82799	2.15E-11	8.67E-11
hsa-mir-541	2.962374	0.512543	22.40536	2.21E-06	5.32E-06
hsa-mir-552	1.619614	4.123391	6.594519	0.010229	0.015344
hsa-mir-577	3.543105	2.891811	54.80158	1.33E-13	6.55E-13
hsa-mir-625	1.286054	8.088431	56.358	6.04E-14	3.02E-13
hsa-mir-675	4.040679	6.028141	45.71681	1.37E-11	5.86E-11
hsa-mir-7-1	1.479855	4.383523	84.26684	4.32E-20	3.15E-19
hsa-mir-708	2.22976	6.731132	111.2344	5.26E-26	5.46E-25
hsa-mir-766	1.486796	3.356044	74.4603	6.19E-18	3.88E-17
hsa-mir-767	6.947759	4.674975	34.74897	3.75E-09	1.22E-08
hsa-mir-889	1.580589	4.319785	22.7041	1.89E-06	4.60E-06
hsa-mir-891a	2.031389	3.631831	16.23902	5.58E-05	0.000115
hsa-mir-9-1	4.63755	10.6544	63.68097	1.46E-15	7.90E-15
hsa-mir-9-2	4.644384	10.65196	63.39866	1.69E-15	8.94E-15
hsa-mir-92b	1.359489	7.712856	74.4866	6.11E-18	3.88E-17
hsa-mir-935	1.76685	1.568214	16.82096	4.11E-05	8.80E-05
hsa-mir-937	2.959685	2.902378	66.51703	3.47E-16	1.99E-15
hsa-mir-944	1.051065	2.2746	8.094257	0.004441	0.006971
hsa-mir-96	2.013587	4.618347	94.50254	2.45E-22	2.07E-21
hsa-mir-99a	-1.22028	8.368973	73.40783	1.05E-17	6.47E-17

Table S2. Clinical covariates for the TCGA LUAD cohort by miRNA signature group

**Covariates**		**Total**	**High-risk group**	**Low-risk group**	***P*-value**
Age, years, no (%)	<=65	174 (46.8)	83 (46.4)	91 (47.2)	0.9625
	>65	198 (53.2)	96 (53.6)	102 (52.8)	
Gender, no (%)	Male	176 (47.3)	87 (48.6)	89 (46.1)	0.6308
	Female	196 (52.7)	92 (51.4)	104 (53.9)	
Disease Stage, no (%)	I	199 (53.5)	83 (46.4)	116 (60.1)	0. 0252
	II	89 (23.9)	44 (24.6)	45 (23.3)	
	III	66 (17.7)	40 (22.3)	26 (13.5)	
	IV	17 (4.6)	11 (6.1)	6 (3.1)	
Lymph Node Involvement,	N0	231 (62.1)	95 (53.1)	136 (70.5)	0.0085
no (%)	N1	72 (19.4)	40 (22.3)	32 (16.6)	
	N2	58 (15.6)	36 (20.1)	22 (11.4)	
	N3	1 (0.3)	1 (0.6)	0 (0.0)	

M stage, no (%)	M0	266 (71.5)	134 (74.9)	132 (68.4)	0.1532
	M1	16 (4.3)	11 (6.1)	5 (2.6)	
T stage, no (%)	T1	112 (30.1)	42 (23.5)	60 (31.1)	0.6659
	T2	211 (56.7)	102 (57.0)	109 (56.5)	
	T3	31 (8.3)	15 (8.4)	16 (8.3)	
	T4	16 (4.3)	8 (4.5)	8 (4.1)	
Smoking status no (%)	Nonsmoker	143 (38.4)	66 (36.9)	77 (39.9)	0.6780
	Smoker	217 (58.3)	105 (58.7)	112 (58.0)	

*Nonsmoker is who did not smoke in his/her lifetime, or reformed smoking more than 15 years.

Table S3. Clinical covariates for the TCGA LUAD cohort by smoking status

Covariates		Total	Nonsmoker	Smoker	*P*-value
Age, years, no (%)	<=65	174 (46.8)	44 (30.8)	126 (58.1)	0.000
	>65	198 (53.2)	99 (69.2)	91 (41.9)	
Gender, no (%)	Male	176 (47.3)	64 (44.8)	107 (49.3)	0.3972
	Female	196 (52.7)	79 (55.2)	110 (50.7)	
Disease Stage, no (%)	I	199 (53.5)	79 (55.2)	116 (53.5)	0.9657
	II	89 (23.9)	33 (23.1)	52 (24.0)	
	III	66 (17.7)	23 (16.1)	39 (18.0)	
	IV	17 (4.6)	7 (4.9)	10 (4.6)	
Lymph Node Involvement, no (%)	N0	231 (62.1)	91 (6.36)	137 (63.1)	0.7485
	N1	72 (19.4)	23 (16.1)	43 (19.8)	
	N2	58 (15.6)	22 (15.4)	33 (15.2)	
	N3	1 (0.3)	0 (0.0)	1 (0.5)	
M stage, no (%)	M0	266 (71.5)	101 (70.6)	154 (71.0)	0.8671
	M1	16 (4.3)	6 (4.2)	10 (4.6)	
T stage, no (%)	T1	112 (30.1)	42 (29.4)	67 (30.9)	0.5311
	T2	211 (56.7)	84 (58.7)	120 (55.3)	
	T3	31 (8.3)	12 (8.)	19 (8.8)	
	T4	16 (4.3)	3 (2.1)	11 (5.1)	

*Nonsmoker is who did not smoke in his/her lifetime, or reformed smoking more than 15 years.

Table S4. Multivariate Cox proportional hazards analysis

Covariates	Coef	HR	Lower 95%CI of HR	Upper 95%CI of HR	*P*-value
age	0.020	1.020	0.998	1.043	0.082
T stage	0.386	1.470	0.835	2.588	0.182
N stage	0.958	2.607	1.730	3.929	4.66e-06
M stage	0.543	1.723	0.838	3.538	0.139
8-miRNA signature	0.401	1.493	1.251	1.782	8.83e-06

Table S5. The over-representation analysis for target genes

Category	Subcategory	Subcategory alternative name	Expected	Observed	*P*-value (FDR)	Enrichment
KEGG	Olfactory transduction	4740	152.975	24	2.80E-52	down
KEGG	Axon guidance	4360	50.8601	87	7.27E-09	up
KEGG	Systemic lupus erythematosus	5322	55.5913	21	7.81E-09	down
KEGG	**Focal adhesion**	4510	79.2472	118	7.43E-07	up
KEGG	**Pathways in cancer**	5200	129.319	177	1.14E-06	up
KEGG	**Wnt signaling pathway**	4310	59.534	92	1.76E-06	up
KEGG	Ribosome	3010	34.6953	13	1.03E-05	down
KEGG	Parkinson's disease	5012	52.0429	26	1.93E-05	down
KEGG	Oxidative phosphorylation	190	52.8315	27	2.58E-05	down
KEGG	Insulin signaling pathway	4910	54.0142	81	3.86E-05	up
KEGG	Neurotrophin signaling pathway	4722	49.6773	75	5.94E-05	up
KEGG	**Adherens junction**	4520	29.5698	48	0.000222	up
KEGG	Regulation of actin cytoskeleton	4810	85.1611	115	0.000301	up
KEGG	Proteasome	3050	18.9247	6	0.000574	down
KEGG	Melanogenesis	4916	40.215	60	0.000687	up
KEGG	Adipocytokine signaling pathway	4920	26.4157	42	0.001007	up
KEGG	Chronic myeloid leukemia	5220	28.7813	45	0.001007	up
KEGG	Glioma	5214	25.6272	41	0.001007	up
KEGG	Prostate cancer	5215	35.0895	53	0.001007	up
KEGG	T cell receptor signaling pathway	4660	42.5806	62	0.001056	up
KEGG	**ErbB signaling pathway**	4012	34.301	51	0.002005	up
KEGG	Endometrial cancer	5213	20.5018	33	0.003361	up
KEGG	**Non-small cell lung cancer**	5223	21.2903	34	0.003361	up
KEGG	**Apoptosis**	4210	34.6953	50	0.005584	up
KEGG	Arachidonic acid metabolism	590	22.8673	11	0.005584	down
KEGG	Calcium signaling pathway	4020	70.1791	91	0.006491	up
KEGG	Endocytosis	4144	80.8242	103	0.006491	up
KEGG	Huntington's disease	5016	72.5447	52	0.006491	down
KEGG	Drug metabolism - cytochrome P450	982	28.7813	16	0.007801	down
KEGG	Gap junction	4540	35.4838	50	0.008649	up
KEGG	Melanoma	5218	27.9928	41	0.008649	up
KEGG	Spliceosome	3040	50.4659	34	0.009061	down
KEGG	**Small cell lung cancer**	5222	33.1182	47	0.009182	up
KEGG	Basal cell carcinoma	5217	21.6846	33	0.009326	up
KEGG	Metabolism of xenobiotics by cytochrome P450	980	27.9928	16	0.011024	down
KEGG	Cell adhesion molecules (CAMs)	4514	53.2257	70	0.011676	up
KEGG	Leukocyte transendothelial migration	4670	46.5232	62	0.013309	up

KEGG	SNARE interactions in vesicular transport	4130	14.1935	23	0.01363	up
KEGG	**p53 signaling pathway**	4115	27.2043	39	0.014989	up
KEGG	**MAPK signaling pathway**	4010	105.269	127	0.017525	up
KEGG	Gastric acid secretion	4971	29.1756	41	0.018487	up
KEGG	Thyroid cancer	5216	11.4337	19	0.019022	up
KEGG	Type II diabetes mellitus	4930	18.5304	28	0.019022	up
KEGG	Lysosome	4142	47.706	62	0.023843	up
KEGG	Amyotrophic lateral sclerosis (ALS)	5014	20.896	30	0.035011	up
KEGG	Chemokine signaling pathway	4062	74.516	91	0.035011	up
KEGG	Fatty acid elongation in mitochondria	62	3.15412	7	0.035011	up
KEGG	Long-term potentiation	4720	27.5985	38	0.035011	up
KEGG	Ubiquitin mediated proteolysis	4120	54.8028	69	0.035662	up
KEGG	Colorectal cancer	5210	24.4444	34	0.039649	up
KEGG	ECM-receptor interaction	4512	33.1182	44	0.041892	up
KEGG	Glycosphingolipid biosynthesis - lacto and neolacto series	601	10.6451	17	0.044412	up
KEGG	Phosphatidylinositol signaling system	4070	30.7526	41	0.047252	up
KEGG	Autoimmune thyroid disease	5320	21.2903	13	0.048008	down
KEGG	VEGF signaling pathway	4370	29.9641	40	0.048008	up
Gene Ontology	olfactory receptor activity	GO:0004984	160.616	5	4.24E-76	down
Gene Ontology	sensory perception of chemical stimulus	GO:0007606	183.232	19	3.67E-69	down
Gene Ontology	sensory perception of smell	GO:0007608	165.599	13	1.57E-67	down
Gene Ontology	cellular process	GO:0009987	4455.08	4935	2.83E-52	up
Gene Ontology	binding	GO:0005488	4747.18	5170	1.94E-43	up
Gene Ontology	biological regulation	GO:0065007	2672.2	3112	8.76E-41	up
Gene Ontology	G-protein coupled receptor activity	GO:0004930	327.748	150	1.05E-38	down
Gene Ontology	regulation of biological process	GO:0050789	2520.79	2933	1.31E-36	up
Gene Ontology	regulation of cellular process	GO:0050794	2395.82	2795	4.49E-35	up
Gene Ontology	protein binding	GO:0005515	3122.62	3531	6.20E-34	up
Gene Ontology	signaling	GO:0023052	1368.88	1663	1.17E-26	up
Gene Ontology	anatomical structure development	GO:0048856	1093.64	1350	5.24E-24	up
Gene Ontology	sensory perception	GO:0007600	318.165	178	6.79E-24	down
Gene Ontology	developmental process	GO:0032502	1311.76	1582	2.92E-23	up
Gene Ontology	system development	GO:0048731	986.312	1228	2.92E-23	up
Gene Ontology	intracellular	GO:0005622	4342.76	4665	8.87E-23	up
Gene Ontology	signaling pathway	GO:0023033	976.345	1210	6.14E-22	up
Gene Ontology	multicellular organismal development	GO:0007275	1195.61	1447	1.25E-21	up
Gene Ontology	localization	GO:0051179	1288.76	1542	1.00E-20	up

Gene Ontology	cellular macromolecule metabolic process	GO:0044260	2184.61	2476	1.83E-19	up
Gene Ontology	nervous system development	GO:0007399	450.031	606	2.99E-19	up
Gene Ontology	signal transmission	GO:0023060	985.545	1205	3.07E-19	up
Gene Ontology	signaling process	GO:0023046	987.845	1207	3.56E-19	up
Gene Ontology	cellular metabolic process	GO:0044237	2848.54	3153	3.56E-19	up
Gene Ontology	membrane-bounded organelle	GO:0043227	3213.47	3519	6.66E-19	up
Gene Ontology	intracellular membrane-bounded organelle	GO:0043231	3210.78	3516	7.01E-19	up
Gene Ontology	intracellular part	GO:0044424	4207.45	4503	8.38E-19	up
Gene Ontology	plasma membrane part	GO:0044459	775.48	970	1.46E-18	up
Gene Ontology	regulation of metabolic process	GO:0019222	1514.54	1764	5.54E-18	up
Gene Ontology	regulation of cellular metabolic process	GO:0031323	1444.39	1688	9.61E-18	up
Gene Ontology	primary metabolic process	GO:0044238	2924.44	3216	1.58E-17	up
Gene Ontology	cytoplasm	GO:0005737	2942.45	3231	3.82E-17	up
Gene Ontology	cognition	GO:0050890	355.732	230	4.47E-17	down
Gene Ontology	intracellular signaling pathway	GO:0023034	583.43	747	7.03E-17	up
Gene Ontology	regulation of primary metabolic process	GO:0080090	1374.24	1607	7.78E-17	up
Gene Ontology	organ development	GO:0048513	740.98	922	8.85E-17	up
Gene Ontology	macromolecule metabolic process	GO:0043170	2405.02	2680	1.01E-16	up
Gene Ontology	cellular developmental process	GO:0048869	737.53	916	2.17E-16	up
Gene Ontology	cell communication	GO:0007154	646.68	814	3.65E-16	up
Gene Ontology	regulation of biosynthetic process	GO:0009889	1176.83	1390	6.50E-16	up
Gene Ontology	signal transduction	GO:0007165	855.596	1042	9.03E-16	up
Gene Ontology	transmembrane receptor activity	GO:0004888	488.748	348	9.42E-16	down
Gene Ontology	regulation of cellular biosynthetic process	GO:0031326	1168.39	1379	1.21E-15	up
Gene Ontology	regulation of macromolecule metabolic process	GO:0060255	1307.54	1527	1.35E-15	up
Gene Ontology	intracellular organelle	GO:0043229	3582.62	3858	1.63E-15	up
Gene Ontology	organelle	GO:0043226	3587.98	3862	2.28E-15	up
Gene Ontology	cell differentiation	GO:0030154	703.03	871	3.36E-15	up
Gene Ontology	anatomical structure morphogenesis	GO:0009653	535.131	683	5.23E-15	up
Gene Ontology	regulation of nucleobase, nucleoside, nucleotide and nucleic acid metabolic process	GO:0019219	1165.33	1370	7.62E-15	up
Gene Ontology	biological_process	GO:0008150	5488.54	5700	9.45E-15	up
Gene Ontology	cellular biosynthetic process	GO:0044249	1658.29	1890	9.85E-15	up
Gene Ontology	molecular_function	GO:0003674	5927.07	6106	1.07E-14	up
Gene Ontology	regulation of nitrogen compound metabolic process	GO:0051171	1175.29	1379	1.23E-14	up

Gene Ontology	regulation of macromolecule biosynthetic process	GO:0010556	1112.04	1311	1.37E-14	up
Gene Ontology	positive regulation of biological process	GO:0048518	858.662	1036	2.52E-14	up
Gene Ontology	biosynthetic process	GO:0009058	1700.46	1929	3.99E-14	up
Gene Ontology	metabolic process	GO:0008152	3212.7	3474	5.21E-14	up
Gene Ontology	intracellular signal transduction	GO:0035556	461.914	594	9.66E-14	up
Gene Ontology	positive regulation of cellular process	GO:0048522	781.23	947	1.40E-13	up
Gene Ontology	regulation of transcription	GO:0045449	1014.68	1198	2.36E-13	up
Gene Ontology	regulation of gene expression	GO:0010468	1133.89	1325	2.77E-13	up
Gene Ontology	transcription regulator activity	GO:0030528	583.814	728	2.83E-13	up
Gene Ontology	establishment of localization	GO:0051234	1115.49	1303	5.81E-13	up
Gene Ontology	cell part	GO:0044464	5775.27	5954	7.56E-13	up
Gene Ontology	cell	GO:0005623	5775.66	5954	8.41E-13	up
Gene Ontology	nucleobase, nucleoside, nucleotide and nucleic acid metabolic process	GO:0006139	1646.03	1860	1.06E-12	up
Gene Ontology	transcription	GO:0006350	1051.86	1233	1.06E-12	up
Gene Ontology	cell surface receptor linked signaling pathway	GO:0007166	573.847	713	1.39E-12	up
Gene Ontology	positive regulation of gene expression	GO:0010628	242.265	334	3.82E-12	up
Gene Ontology	transport	GO:0006810	1101.69	1281	5.34E-12	up
Gene Ontology	cellular macromolecule biosynthetic process	GO:0034645	1352.78	1545	8.82E-12	up
Gene Ontology	regulation of cell communication	GO:0010646	449.648	570	9.46E-12	up
Gene Ontology	macromolecule biosynthetic process	GO:0009059	1378.84	1572	9.68E-12	up
Gene Ontology	protein modification process	GO:0006464	677.73	822	1.07E-11	up
Gene Ontology	positive regulation of macromolecule biosynthetic process	GO:0010557	269.099	363	1.51E-11	up
Gene Ontology	positive regulation of nucleobase, nucleoside, nucleotide and nucleic acid metabolic process	GO:0045935	260.665	353	1.64E-11	up
Gene Ontology	nitrogen compound metabolic process	GO:0006807	1800.89	2010	1.77E-11	up
Gene Ontology	positive regulation of cellular biosynthetic process	GO:0031328	285.965	382	2.02E-11	up
Gene Ontology	cellular nitrogen compound metabolic process	GO:0034641	1757.19	1963	2.66E-11	up
Gene Ontology	cellular component organization	GO:0016043	1076.39	1248	3.06E-11	up
Gene Ontology	positive regulation of biosynthetic	GO:0009891	290.182	386	3.15E-11	up

	process					
Gene Ontology	positive regulation of transcription	GO:0045941	229.616	315	3.97E-11	up
Gene Ontology	cytoplasmic part	GO:0044444	1987.19	2199	3.99E-11	up
Gene Ontology	regulation of signaling pathway	GO:0035466	391.765	501	4.83E-11	up
Gene Ontology	positive regulation of nitrogen compound metabolic process	GO:0051173	268.715	360	5.67E-11	up
Gene Ontology	macromolecule localization	GO:0033036	481.464	601	5.91E-11	up
Gene Ontology	macromolecule modification	GO:0043412	708.397	849	9.58E-11	up
Gene Ontology	transcription, DNA-dependent	GO:0006351	747.113	890	1.34E-10	up
Gene Ontology	regulation of transcription, DNA-dependent	GO:0006355	697.663	835	2.15E-10	up
Gene Ontology	RNA biosynthetic process	GO:0032774	748.646	890	2.31E-10	up
Gene Ontology	regulation of RNA metabolic process	GO:0051252	716.446	854	3.39E-10	up
Gene Ontology	negative regulation of cellular process	GO:0048523	722.963	861	3.42E-10	up
Gene Ontology	nucleic acid metabolic process	GO:0090304	1427.53	1608	4.56E-10	up
Gene Ontology	post-translational protein modification	GO:0043687	578.447	702	6.05E-10	up
Gene Ontology	neurogenesis	GO:0022008	247.632	331	6.39E-10	up
Gene Ontology	small GTPase mediated signal transduction	GO:0007264	165.983	234	1.14E-09	up
Gene Ontology	metal ion binding	GO:0046872	1451.29	1628	1.51E-09	up
Gene Ontology	central nervous system development	GO:0007417	177.482	247	1.76E-09	up
Gene Ontology	regulation of transcription from RNA polymerase II promoter	GO:0006357	291.332	379	2.01E-09	up
Gene Ontology	insoluble fraction	GO:0005626	318.165	409	2.56E-09	up
Gene Ontology	intrinsic to plasma membrane	GO:0031226	463.064	571	2.65E-09	up
Gene Ontology	endomembrane system	GO:0012505	556.981	674	2.96E-09	up
Gene Ontology	cation binding	GO:0043169	1466.63	1641	3.00E-09	up
Gene Ontology	positive regulation of transcription, DNA-dependent	GO:0045893	197.799	270	3.04E-09	up
Gene Ontology	vasculature development	GO:0001944	133.399	193	3.41E-09	up
Gene Ontology	transcription factor activity	GO:0003700	369.532	466	3.60E-09	up
Gene Ontology	regulation of biological quality	GO:0065008	660.48	786	3.60E-09	up
Gene Ontology	ion binding	GO:0043167	1470.46	1644	3.65E-09	up
Gene Ontology	enzyme linked receptor protein signaling pathway	GO:0007167	182.082	250	7.22E-09	up
Gene Ontology	cellular component movement	GO:0006928	250.315	329	8.50E-09	up
Gene Ontology	purine nucleotide binding	GO:0017076	741.363	870	1.01E-08	up
Gene Ontology	integral to plasma membrane	GO:0005887	454.631	558	1.03E-08	up
Gene Ontology	transcription from RNA polymerase II promoter	GO:0006366	352.282	444	1.10E-08	up

Gene Ontology	ribonucleotide binding	GO:0032553	709.547	835	1.24E-08	up
Gene Ontology	purine ribonucleotide binding	GO:0032555	709.547	835	1.24E-08	up
Gene Ontology	positive regulation of RNA metabolic process	GO:0051254	200.099	270	1.31E-08	up
Gene Ontology	cell migration	GO:0016477	177.099	243	1.33E-08	up
Gene Ontology	Golgi apparatus	GO:0005794	342.315	432	1.51E-08	up
Gene Ontology	blood vessel development	GO:0001568	130.333	187	1.52E-08	up
Gene Ontology	receptor activity	GO:0004872	647.064	530	1.69E-08	down
Gene Ontology	organ morphogenesis	GO:0009887	252.615	330	1.79E-08	up
Gene Ontology	positive regulation of macromolecule metabolic process	GO:0010604	374.132	467	1.88E-08	up
Gene Ontology	gland development	GO:0048732	78.1996	122	2.19E-08	up
Gene Ontology	cell surface	GO:0009986	134.166	191	2.25E-08	up
Gene Ontology	positive regulation of transcription from RNA polymerase II promoter	GO:0045944	152.566	213	2.27E-08	up
Gene Ontology	protein localization	GO:0008104	401.731	497	2.43E-08	up
Gene Ontology	cytoskeletal protein binding	GO:0008092	196.266	264	2.86E-08	up
Gene Ontology	negative regulation of biological process	GO:0048519	791.196	919	3.38E-08	up
Gene Ontology	adenyl nucleotide binding	GO:0030554	607.197	721	3.42E-08	up
Gene Ontology	phosphorus metabolic process	GO:0006793	501.398	605	4.54E-08	up
Gene Ontology	phosphate metabolic process	GO:0006796	501.398	605	4.54E-08	up
Gene Ontology	purine nucleoside binding	GO:0001883	620.997	735	4.55E-08	up
Gene Ontology	nucleoside binding	GO:0001882	624.83	739	4.73E-08	up
Gene Ontology	protein amino acid phosphorylation	GO:0006468	309.348	392	4.97E-08	up
Gene Ontology	adenyl ribonucleotide binding	GO:0032559	576.147	686	5.10E-08	up
Gene Ontology	generation of neurons	GO:0048699	230.382	302	5.75E-08	up
Gene Ontology	membrane fraction	GO:0005624	306.282	388	6.22E-08	up
Gene Ontology	zinc ion binding	GO:0008270	770.113	894	6.49E-08	up
Gene Ontology	DNA binding	GO:0003677	898.912	1031	6.76E-08	up
Gene Ontology	transferase activity, transferring phosphorus-containing groups	GO:0016772	343.082	429	6.76E-08	up
Gene Ontology	transmembrane receptor protein tyrosine kinase signaling pathway	GO:0007169	113.466	164	7.85E-08	up
Gene Ontology	regulation of signal transduction	GO:0009966	302.449	383	8.16E-08	up
Gene Ontology	positive regulation of metabolic process	GO:0009893	403.265	495	9.29E-08	up
Gene Ontology	cell motility	GO:0048870	189.749	254	9.71E-08	up
Gene Ontology	localization of cell	GO:0051674	189.749	254	9.71E-08	up
Gene Ontology	positive regulation of cellular metabolic process	GO:0031325	382.948	472	1.12E-07	up
Gene Ontology	regulation of signaling process	GO:0023051	304.365	384	1.31E-07	up
Gene Ontology	neuron differentiation	GO:0030182	209.299	276	1.32E-07	up

Gene Ontology	kinase activity	GO:0016301	294.782	373	1.44E-07	up
Gene Ontology	ATP binding	GO:0005524	567.331	673	1.44E-07	up
Gene Ontology	nucleus	GO:0005634	1992.56	2165	1.50E-07	up
Gene Ontology	transition metal ion binding	GO:0046914	875.912	1003	1.66E-07	up
Gene Ontology	phosphotransferase activity, alcohol group as acceptor	GO:0016773	271.399	346	1.87E-07	up
Gene Ontology	**cell junction**	GO:0030054	206.616	272	2.04E-07	up
Gene Ontology	regulation of localization	GO:0032879	284.049	360	2.08E-07	up
Gene Ontology	**Ras protein signal transduction**	GO:0007265	87.7829	131	2.20E-07	up
Gene Ontology	enzyme binding	GO:0019899	245.715	316	2.80E-07	up
Gene Ontology	tissue development	GO:0009888	317.015	396	3.09E-07	up
Gene Ontology	anatomical structure formation involved in morphogenesis	GO:0048646	180.932	241	4.06E-07	up
Gene Ontology	**Wnt receptor signaling pathway**	GO:0016055	72.4496	111	4.35E-07	up
Gene Ontology	transmembrane receptor protein kinase activity	GO:0019199	31.4332	57	4.55E-07	up
Gene Ontology	cell fraction	GO:0000267	412.848	501	4.56E-07	up
Gene Ontology	**cell adhesion**	GO:0007155	317.015	395	4.56E-07	up
Gene Ontology	cell development	GO:0048468	308.198	385	4.84E-07	up
Gene Ontology	nucleotide binding	GO:0000166	865.179	987	5.26E-07	up
Gene Ontology	embryonic development	GO:0009790	239.965	308	5.30E-07	up
Gene Ontology	biological adhesion	GO:0022610	317.398	395	5.30E-07	up
Gene Ontology	tissue morphogenesis	GO:0048729	111.933	159	5.92E-07	up
Gene Ontology	urogenital system development	GO:0001655	56.3497	90	6.41E-07	up
Gene Ontology	cell morphogenesis	GO:0000902	164.833	221	7.82E-07	up
Gene Ontology	regulation of cellular component organization	GO:0051128	211.982	275	9.06E-07	up
Gene Ontology	organelle membrane	GO:0031090	729.863	841	9.20E-07	up
Gene Ontology	cell projection organization	GO:0030030	175.566	233	9.81E-07	up
Gene Ontology	morphogenesis of an epithelium	GO:0002009	85.4829	126	1.05E-06	up
Gene Ontology	synapse	GO:0045202	142.216	194	1.05E-06	up
Gene Ontology	regulation of developmental process	GO:0050793	309.348	384	1.16E-06	up
Gene Ontology	cell proliferation	GO:0008283	447.731	536	1.29E-06	up
Gene Ontology	heart development	GO:0007507	93.1495	135	1.37E-06	up
Gene Ontology	cell projection	GO:0042995	293.632	366	1.39E-06	up
Gene Ontology	basolateral plasma membrane	GO:0016323	85.8662	126	1.46E-06	up
Gene Ontology	protein domain specific binding	GO:0019904	150.649	203	1.69E-06	up
Gene Ontology	defense response to bacterium	GO:0042742	46.3831	19	1.72E-06	down
Gene Ontology	brain development	GO:0007420	121.899	169	1.88E-06	up
Gene Ontology	cellular response to stimulus	GO:0051716	422.431	507	2.01E-06	up
Gene Ontology	transcription activator activity	GO:0016563	164.833	219	2.15E-06	up
Gene Ontology	**cell-cell signaling**	GO:0007267	275.999	345	2.31E-06	up
Gene Ontology	Golgi apparatus part	GO:0044431	206.999	267	2.54E-06	up

Gene Ontology	blood vessel morphogenesis	GO:0048514	113.083	158	2.54E-06	up
Gene Ontology	embryonic morphogenesis	GO:0048598	133.399	182	2.58E-06	up
Gene Ontology	cellular protein metabolic process	GO:0044267	944.912	1064	2.77E-06	up
Gene Ontology	locomotion	GO:0040011	236.516	300	3.04E-06	up
Gene Ontology	endosome	GO:0005768	148.349	199	3.34E-06	up
Gene Ontology	membrane	GO:0016020	2792.95	2960	3.50E-06	up
Gene Ontology	RNA metabolic process	GO:0016070	995.128	1115	4.12E-06	up
Gene Ontology	**canonical Wnt receptor signaling pathway**	GO:0060070	30.6665	54	4.38E-06	up
Gene Ontology	regulation of apoptosis	GO:0042981	340.782	415	4.56E-06	up
Gene Ontology	receptor signaling protein activity	GO:0005057	61.333	94	4.59E-06	up
Gene Ontology	regulation of cell death	GO:0010941	346.532	421	5.10E-06	up
Gene Ontology	regulation of cell proliferation	GO:0042127	326.598	399	5.14E-06	up
Gene Ontology	regulation of programmed cell death	GO:0043067	343.848	418	5.14E-06	up
Gene Ontology	Golgi membrane	GO:0000139	172.499	226	5.16E-06	up
Gene Ontology	neuron projection	GO:0043005	142.216	191	5.16E-06	up
Gene Ontology	cellular component morphogenesis	GO:0032989	181.316	236	5.41E-06	up
Gene Ontology	transmission of nerve impulse	GO:0019226	161.383	213	5.69E-06	up
Gene Ontology	membrane organization	GO:0061024	170.966	224	5.74E-06	up
Gene Ontology	neurological system process	GO:0050877	487.598	403	6.45E-06	down
Gene Ontology	transferase activity	GO:0016740	648.213	746	6.81E-06	up
Gene Ontology	intermediate filament cytoskeleton	GO:0045111	71.683	39	7.12E-06	down
Gene Ontology	negative regulation of transcription, DNA-dependent	GO:0045892	158.316	209	7.12E-06	up
Gene Ontology	cellular localization	GO:0051641	462.298	546	7.40E-06	up
Gene Ontology	cellular membrane organization	GO:0016044	170.582	223	7.42E-06	up
Gene Ontology	regulation of cell differentiation	GO:0045595	215.816	274	9.03E-06	up
Gene Ontology	growth	GO:0040007	206.999	264	9.16E-06	up
Gene Ontology	gene expression	GO:0010467	1458.96	1593	9.81E-06	up
Gene Ontology	RNA polymerase II transcription factor activity	GO:0003702	91.6162	130	1.02E-05	up
Gene Ontology	cell-substrate adhesion	GO:0031589	58.2664	89	1.11E-05	up
Gene Ontology	active transmembrane transporter activity	GO:0022804	131.483	177	1.11E-05	up
Gene Ontology	intermediate filament	GO:0005882	68.233	37	1.20E-05	down
Gene Ontology	external side of plasma membrane	GO:0009897	53.6664	83	1.30E-05	up
Gene Ontology	phosphorylation	GO:0016310	437.381	517	1.34E-05	up
Gene Ontology	establishment of protein localization	GO:0045184	346.532	418	1.35E-05	up
Gene Ontology	cell-matrix adhesion	GO:0007160	45.9998	73	1.57E-05	up
Gene Ontology	skeletal system development	GO:0001501	146.816	194	1.66E-05	up
Gene Ontology	mitochondrial respiratory chain	GO:0005746	26.8332	8	1.68E-05	down

Gene Ontology	cell morphogenesis involved in differentiation	GO:0000904	116.916	159	1.86E-05	up
Gene Ontology	negative regulation of RNA metabolic process	GO:0051253	160.999	210	1.91E-05	up
Gene Ontology	morphogenesis of a branching structure	GO:0001763	50.9831	79	2.13E-05	up
Gene Ontology	protein kinase activity	GO:0004672	227.699	285	2.34E-05	up
Gene Ontology	regulation of multicellular organismal process	GO:0051239	416.298	492	2.54E-05	up
Gene Ontology	morphogenesis of a branching epithelium	GO:0061138	43.3165	69	2.54E-05	up
Gene Ontology	respiratory chain	GO:0070469	29.5165	10	2.58E-05	down
Gene Ontology	kidney development	GO:0001822	41.0165	66	2.59E-05	up
Gene Ontology	vesicle-mediated transport	GO:0016192	257.599	318	2.62E-05	up
Gene Ontology	translational elongation	GO:0006414	39.8665	17	2.66E-05	down
Gene Ontology	neuron development	GO:0048666	152.949	200	2.71E-05	up
Gene Ontology	neuron projection morphogenesis	GO:0048812	94.6829	132	2.79E-05	up
Gene Ontology	anchoring junction	GO:0070161	62.483	93	2.80E-05	up
Gene Ontology	plasma membrane	GO:0005886	1440.94	1568	2.97E-05	up
Gene Ontology	forebrain development	GO:0030900	66.6997	98	3.22E-05	up
Gene Ontology	ribosomal subunit	GO:0033279	46.3831	22	3.85E-05	down
Gene Ontology	renal system development	GO:0072001	42.1665	67	3.96E-05	up
Gene Ontology	actin cytoskeleton organization	GO:0030036	108.866	148	4.31E-05	up
Gene Ontology	negative regulation of transcription	GO:0016481	196.266	248	4.60E-05	up
Gene Ontology	adherens junction	GO:0005912	55.9664	84	5.67E-05	up
Gene Ontology	cell morphogenesis involved in neuron differentiation	GO:0048667	92.3829	128	6.00E-05	up
Gene Ontology	catalytic activity	GO:0003824	1992.56	2129	6.39E-05	up
Gene Ontology	cellular response to organic substance	GO:0071310	103.499	141	6.39E-05	up
Gene Ontology	regulation of cellular component movement	GO:0051270	94.2995	130	7.00E-05	up
Gene Ontology	negative regulation of cellular biosynthetic process	GO:0031327	241.115	297	7.09E-05	up
Gene Ontology	neuron projection development	GO:0031175	123.433	164	7.21E-05	up
Gene Ontology	sequence-specific DNA binding	GO:0043565	248.399	305	7.24E-05	up
Gene Ontology	cell leading edge	GO:0031252	64.3997	94	7.42E-05	up
Gene Ontology	negative regulation of macromolecule biosynthetic process	GO:0010558	234.216	289	7.97E-05	up
Gene Ontology	extracellular region	GO:0005576	780.463	687	8.47E-05	down
Gene Ontology	chordate embryonic development	GO:0043009	143.749	187	8.59E-05	up
Gene Ontology	cellular response to endogenous stimulus	GO:0071495	72.833	104	8.66E-05	up

Gene Ontology	**apoptosis**	GO:0006915	434.315	507	8.85E-05	up
Gene Ontology	ribonucleoprotein complex	GO:0030529	193.199	145	9.18E-05	down
Gene Ontology	cell death	GO:0008219	479.164	555	9.26E-05	up
Gene Ontology	programmed cell death	GO:0012501	437.381	510	9.54E-05	up
Gene Ontology	regulation of phosphate metabolic process	GO:0019220	213.132	265	9.54E-05	up
Gene Ontology	regulation of phosphorus metabolic process	GO:0051174	213.132	265	9.54E-05	up
Gene Ontology	cytoskeleton organization	GO:0007010	202.399	253	9.54E-05	up
Gene Ontology	synaptic transmission	GO:0007268	138.766	181	9.61E-05	up
Gene Ontology	death	GO:0016265	480.314	556	9.66E-05	up
Gene Ontology	cyclic-nucleotide phosphodiesterase activity	GO:0004112	9.58329	21	0.000102	up
Gene Ontology	negative regulation of biosynthetic process	GO:0009890	244.949	300	0.000107	up
Gene Ontology	intracellular protein kinase cascade	GO:0007243	228.849	282	0.000112	up
Gene Ontology	signal transmission via phosphorylation event	GO:0023014	228.849	282	0.000112	up
Gene Ontology	DNA regulatory region binding	GO:0044212	47.9164	73	0.000112	up
Gene Ontology	muscle organ development	GO:0007517	98.5162	134	0.000115	up
Gene Ontology	embryonic development ending in birth or egg hatching	GO:0009792	145.283	188	0.000117	up
Gene Ontology	anterior/posterior pattern formation	GO:0009952	59.4164	87	0.000134	up
Gene Ontology	transmembrane receptor protein tyrosine kinase activity	GO:0004714	24.9165	43	0.000136	up
Gene Ontology	growth factor binding	GO:0019838	42.5498	66	0.000136	up
Gene Ontology	axonogenesis	GO:0007409	83.5663	116	0.00014	up
Gene Ontology	negative regulation of cell communication	GO:0010648	129.183	169	0.000153	up
Gene Ontology	mitochondrial membrane part	GO:0044455	50.9831	27	0.000156	down
Gene Ontology	actin filament-based process	GO:0030029	115.383	153	0.000159	up
Gene Ontology	cell projection morphogenesis	GO:0048858	108.483	145	0.000159	up
Gene Ontology	protein transport	GO:0015031	340.398	403	0.000173	up
Gene Ontology	PDZ domain binding	GO:0030165	20.6999	37	0.000174	up
Gene Ontology	cell part morphogenesis	GO:0032990	113.849	151	0.000178	up
Gene Ontology	protein serine/threonine kinase activity	GO:0004674	162.916	207	0.000179	up
Gene Ontology	glycoprotein metabolic process	GO:0009100	79.7329	111	0.00018	up
Gene Ontology	cytoplasmic vesicle	GO:0031410	263.732	319	0.000187	up
Gene Ontology	membrane raft	GO:0045121	58.2664	85	0.000199	up
Gene Ontology	3',5'-cyclic-nucleotide phosphodiesterase activity	GO:0004114	9.19996	20	0.000208	up

Gene Ontology	muscle structure development	GO:0061061	117.683	155	0.000218	up
Gene Ontology	vesicle	GO:0031982	275.232	331	0.000229	up
Gene Ontology	extracellular structure organization	GO:0043062	66.6997	95	0.000231	up
Gene Ontology	**regulation of Wnt receptor signaling pathway**	GO:0030111	35.2665	56	0.000244	up
Gene Ontology	negative regulation of nucleobase, nucleoside, nucleotide and nucleic acid metabolic process	GO:0045934	220.032	270	0.000246	up
Gene Ontology	response to endogenous stimulus	GO:0009719	196.649	244	0.000247	up
Gene Ontology	endoplasmic reticulum	GO:0005783	392.531	458	0.000249	up
Gene Ontology	promoter binding	GO:0010843	46.3831	70	0.000252	up
Gene Ontology	angiogenesis	GO:0001525	90.4662	123	0.000261	up
Gene Ontology	regulation of molecular function	GO:0065009	414.381	481	0.000285	up
Gene Ontology	establishment of localization in cell	GO:0051649	427.798	495	0.000317	up
Gene Ontology	apical part of cell	GO:0045177	79.7329	110	0.000321	up
Gene Ontology	integrin-mediated signaling pathway	GO:0007229	21.8499	38	0.000333	up
Gene Ontology	protein metabolic process	GO:0019538	1125.08	1227	0.000333	up
Gene Ontology	reproductive developmental process	GO:0003006	114.999	151	0.000334	up
Gene Ontology	negative regulation of cell death	GO:0060548	159.083	201	0.000353	up
Gene Ontology	regulation of cell migration	GO:0030334	85.0996	116	0.000384	up
Gene Ontology	positive regulation of cellular component organization	GO:0051130	88.5496	120	0.000394	up
Gene Ontology	endocrine system development	GO:0035270	31.0498	50	0.000399	up
Gene Ontology	endocytosis	GO:0006897	105.799	140	0.000402	up
Gene Ontology	membrane invagination	GO:0010324	105.799	140	0.000402	up
Gene Ontology	dephosphorylation	GO:0016311	67.4663	95	0.000402	up
Gene Ontology	phosphoric ester hydrolase activity	GO:0042578	131.099	169	0.000402	up
Gene Ontology	anaphase-promoting complex-dependent proteasomal ubiquitin-dependent protein catabolic process	GO:0031145	24.5332	9	0.000431	down
Gene Ontology	GTPase regulator activity	GO:0030695	161.383	203	0.000443	up
Gene Ontology	identical protein binding	GO:0042802	267.182	320	0.000449	up
Gene Ontology	cell projection part	GO:0044463	138.383	177	0.000451	up
Gene Ontology	regulation of anatomical structure morphogenesis	GO:0022603	119.983	156	0.000458	up
Gene Ontology	keratin filament	GO:0045095	34.4998	16	0.000458	down
Gene Ontology	reproductive structure development	GO:0048608	65.1663	92	0.000463	up
Gene Ontology	enzyme activator activity	GO:0008047	134.166	172	0.000492	up
Gene Ontology	cytosolic ribosome	GO:0022626	28.7499	12	0.000495	down

Gene Ontology	structural constituent of ribosome	GO:0003735	60.5664	36	0.000498	down
Gene Ontology	SH3 domain binding	GO:0017124	38.3331	59	0.000501	up
Gene Ontology	glycoprotein biosynthetic process	GO:0009101	62.8664	89	0.000536	up
Gene Ontology	negative regulation of cellular metabolic process	GO:0031324	310.115	366	0.000552	up
Gene Ontology	negative regulation of apoptosis	GO:0043066	154.866	195	0.000571	up
Gene Ontology	negative regulation of nitrogen compound metabolic process	GO:0051172	222.332	270	0.000571	up
Gene Ontology	negative regulation of gene expression	GO:0010629	219.649	267	0.000579	up
Gene Ontology	small ribosomal subunit	GO:0015935	22.6166	8	0.000589	down
Gene Ontology	nucleoside-triphosphatase regulator activity	GO:0060589	164.833	206	0.00061	up
Gene Ontology	ruffle	GO:0001726	29.1332	47	0.000616	up
Gene Ontology	negative regulation of cellular component movement	GO:0051271	28.3665	46	0.000617	up
Gene Ontology	homeostatic process	GO:0042592	323.532	380	0.000634	up
Gene Ontology	negative regulation of transcription from RNA polymerase II promoter	GO:0000122	111.933	146	0.000646	up
Gene Ontology	negative regulation of signaling process	GO:0023057	48.2998	71	0.00066	up
Gene Ontology	MAPKKK cascade	GO:0000165	114.616	149	0.000665	up
Gene Ontology	negative regulation of signal transduction	GO:0009968	47.5331	70	0.000686	up
Gene Ontology	regulation of phosphorylation	GO:0042325	203.932	249	0.000716	up
Gene Ontology	ribosome	GO:0005840	75.8996	49	0.000725	down
Gene Ontology	system process	GO:0003008	625.597	551	0.000732	down
Gene Ontology	mitochondrial respiratory chain complex I	GO:0005747	17.6332	5	0.000732	down
Gene Ontology	NADH dehydrogenase complex	GO:0030964	17.6332	5	0.000732	down
Gene Ontology	respiratory chain complex I	GO:0045271	17.6332	5	0.000732	down
Gene Ontology	synapse assembly	GO:0007416	18.0166	32	0.000733	up
Gene Ontology	cell projection assembly	GO:0030031	37.9498	58	0.000733	up
Gene Ontology	fucosyltransferase activity	GO:0008417	5.36664	13	0.000746	up
Gene Ontology	tube development	GO:0035295	130.716	167	0.000753	up
Gene Ontology	regulation of MAPKKK cascade	GO:0043408	65.933	92	0.000775	up
Gene Ontology	regulation of cell morphogenesis	GO:0022604	60.9497	86	0.000798	up
Gene Ontology	perinuclear region of cytoplasm	GO:0048471	126.499	162	0.000827	up
Gene Ontology	leading edge membrane	GO:0031256	20.3166	35	0.000848	up
Gene Ontology	gland morphogenesis	GO:0022612	33.3498	52	0.000851	up
Gene Ontology	transmembrane transporter activity	GO:0022857	346.915	404	0.000861	up
Gene Ontology	negative regulation of programmed cell death	GO:0043069	156.783	196	0.000861	up

Gene Ontology	regulation of locomotion	GO:0040012	94.2995	125	0.000875	up
Gene Ontology	response to organic substance	GO:0010033	346.148	403	0.000897	up
Gene Ontology	developmental growth	GO:0048589	62.8664	88	0.000945	up
Gene Ontology	molecular adaptor activity	GO:0060090	26.4499	43	0.00095	up
Gene Ontology	pattern specification process	GO:0007389	109.249	142	0.000955	up
Gene Ontology	membrane-bounded vesicle	GO:0031988	256.832	306	0.000968	up
Gene Ontology	cytoplasmic membrane-bounded vesicle	GO:0016023	249.549	298	0.000983	up
Gene Ontology	cellular response to hormone stimulus	GO:0032870	69.7663	96	0.001049	up
Gene Ontology	cell-substrate junction	GO:0030055	37.5665	57	0.00108	up
Gene Ontology	T cell activation involved in immune response	GO:0002286	9.19996	19	0.001107	up
Gene Ontology	response to chemical stimulus	GO:0042221	581.13	652	0.00114	up
Gene Ontology	cell-substrate adherens junction	GO:0005924	35.2665	54	0.001185	up
Gene Ontology	regulation of ubiquitin-protein ligase activity involved in mitotic cell cycle	GO:0051439	26.0665	11	0.001216	down
Gene Ontology	in utero embryonic development	GO:0001701	86.2496	115	0.001219	up
Gene Ontology	calmodulin binding	GO:0005516	54.0497	77	0.001225	up
Gene Ontology	beta-catenin binding	GO:0008013	17.6332	31	0.00123	up
Gene Ontology	positive regulation of cell differentiation	GO:0045597	99.2828	130	0.001236	up
Gene Ontology	carboxylesterase activity	GO:0004091	38.3331	20	0.001257	down
Gene Ontology	protein amino acid dephosphorylation	GO:0006470	53.2831	76	0.001278	up
Gene Ontology	guanyl-nucleotide exchange factor activity	GO:0005085	57.4997	81	0.001331	up
Gene Ontology	regulation of transport	GO:0051049	201.249	244	0.001368	up
Gene Ontology	NADH dehydrogenase activity	GO:0003954	16.8666	5	0.001397	down
Gene Ontology	NADH dehydrogenase (ubiquinone) activity	GO:0008137	16.8666	5	0.001397	down
Gene Ontology	NADH dehydrogenase (quinone) activity	GO:0050136	16.8666	5	0.001397	down
Gene Ontology	sensory perception of taste	GO:0050909	16.8666	5	0.001397	down
Gene Ontology	negative regulation of locomotion	GO:0040013	28.3665	45	0.001425	up
Gene Ontology	nucleic acid binding	GO:0003676	1257.71	1354	0.001438	up
Gene Ontology	tubulin binding	GO:0015631	38.7165	58	0.001461	up
Gene Ontology	negative regulation of signaling pathway	GO:0035467	103.116	134	0.001461	up
Gene Ontology	ureteric bud development	GO:0001657	22.9999	38	0.001462	up
Gene Ontology	epithelium development	GO:0060429	142.983	179	0.001517	up
Gene Ontology	regulation of cell projection organization	GO:0031344	50.2164	72	0.001522	up

Gene Ontology	cellular response to stress	GO:0033554	267.565	316	0.001542	up
Gene Ontology	organelle part	GO:0044422	1950.77	2062	0.001564	up
Gene Ontology	synapse part	GO:0044456	105.033	136	0.001579	up
Gene Ontology	response to hormone stimulus	GO:0009725	176.332	216	0.00158	up
Gene Ontology	axon cargo transport	GO:0008088	4.98331	12	0.001606	up
Gene Ontology	secondary active transmembrane transporter activity	GO:0015291	74.7496	101	0.001613	up
Gene Ontology	positive regulation of developmental process	GO:0051094	136.083	171	0.001662	up
Gene Ontology	cellular macromolecule localization	GO:0070727	192.816	234	0.001687	up
Gene Ontology	regulation of protein amino acid phosphorylation	GO:0001932	87.7829	116	0.001725	up
Gene Ontology	epithelial tube morphogenesis	GO:0060562	52.8997	75	0.001743	up
Gene Ontology	cellular protein localization	GO:0034613	192.049	233	0.001771	up
Gene Ontology	**focal adhesion**	GO:0005925	33.3498	51	0.001812	up
Gene Ontology	regulation of cell development	GO:0060284	102.733	133	0.00185	up
Gene Ontology	axon	GO:0030424	68.233	93	0.001958	up
Gene Ontology	organelle organization	GO:0006996	575.381	643	0.001981	up
Gene Ontology	apical plasma membrane	GO:0016324	63.2497	87	0.002111	up
Gene Ontology	central nervous system neuron differentiation	GO:0021953	27.9832	44	0.002164	up
Gene Ontology	negative regulation of cell migration	GO:0030336	26.4499	42	0.002239	up
Gene Ontology	negative regulation of ubiquitin-protein ligase activity involved in mitotic cell cycle	GO:0051436	23.7666	10	0.002262	down
Gene Ontology	protein binding, bridging	GO:0030674	36.7998	55	0.002275	up
Gene Ontology	hexose transport	GO:0008645	22.6166	37	0.002307	up
Gene Ontology	glucose transport	GO:0015758	22.6166	37	0.002307	up
Gene Ontology	metal ion transport	GO:0030001	190.132	230	0.002371	up
Gene Ontology	regionalization	GO:0003002	86.6329	114	0.002372	up
Gene Ontology	intracellular organelle part	GO:0044446	1923.94	2031	0.002433	up
Gene Ontology	intracellular transport	GO:0046907	288.649	337	0.002505	up
Gene Ontology	regulation of cell size	GO:0008361	110.399	141	0.002516	up
Gene Ontology	early endosome	GO:0005769	51.7497	73	0.002538	up
Gene Ontology	phosphoprotein phosphatase activity	GO:0004721	64.3997	88	0.002539	up
Gene Ontology	sex differentiation	GO:0007548	67.8497	92	0.002607	up
Gene Ontology	oxidoreductase activity, acting on NADH or NADPH, quinone or similar compound as acceptor	GO:0016655	19.1666	7	0.002647	down
Gene Ontology	regulation of kinase activity	GO:0043549	150.649	186	0.002647	up
Gene Ontology	cellular response to chemical	GO:0070887	172.499	210	0.002853	up

	stimulus					
Gene Ontology	cellular response to peptide hormone stimulus	GO:0071375	38.7165	57	0.002921	up
Gene Ontology	protein phosphorylated amino acid binding	GO:0045309	5.74997	13	0.002967	up
Gene Ontology	kinase binding	GO:0019900	80.1163	106	0.002977	up
Gene Ontology	nucleoside-triphosphatase activity	GO:0017111	280.982	328	0.003021	up
Gene Ontology	epithelial cell development	GO:0002064	15.3333	27	0.003057	up
Gene Ontology	muscle cell migration	GO:0014812	10.3499	20	0.003057	up
Gene Ontology	diencephalon development	GO:0021536	15.3333	27	0.003057	up
Gene Ontology	substrate-specific transmembrane transporter activity	GO:0022891	315.482	365	0.003095	up
Gene Ontology	branching morphogenesis of a tube	GO:0048754	37.1832	55	0.003136	up
Gene Ontology	synapse organization	GO:0050808	28.3665	44	0.003163	up
Gene Ontology	specific RNA polymerase II transcription factor activity	GO:0003704	16.0999	28	0.003192	up
Gene Ontology	cell growth	GO:0016049	103.883	133	0.003207	up
Gene Ontology	chromatin binding	GO:0003682	68.233	92	0.003257	up
Gene Ontology	regulation of cellular component biogenesis	GO:0044087	60.5664	83	0.003271	up
Gene Ontology	ruffle membrane	GO:0032587	11.1166	21	0.003505	up
Gene Ontology	positive regulation of ubiquitin-protein ligase activity involved in mitotic cell cycle	GO:0051437	24.5332	11	0.003558	down
Gene Ontology	monosaccharide transport	GO:0015749	22.9999	37	0.00356	up
Gene Ontology	negative regulation of Wnt receptor signaling pathway	GO:0030178	22.2332	36	0.003578	up
Gene Ontology	negative regulation of macromolecule metabolic process	GO:0010605	317.015	366	0.003601	up
Gene Ontology	sensory organ development	GO:0007423	96.2162	124	0.00361	up
Gene Ontology	keratinization	GO:0031424	15.7166	5	0.003615	down
Gene Ontology	response to peptide hormone stimulus	GO:0043434	77.0496	102	0.00364	up
Gene Ontology	cell fate commitment	GO:0045165	54.8164	76	0.003641	up
Gene Ontology	regulation of gene-specific transcription from RNA polymerase II promoter	GO:0010551	62.483	85	0.003714	up
Gene Ontology	gene-specific transcription from RNA polymerase II promoter	GO:0032569	62.483	85	0.003714	up
Gene Ontology	muscle tissue development	GO:0060537	65.933	89	0.00379	up
Gene Ontology	regulation of small GTPase mediated signal transduction	GO:0051056	104.266	133	0.003793	up
Gene Ontology	pituitary gland development	GO:0021983	11.8833	22	0.003849	up

Gene Ontology	**negative regulation of canonical Wnt receptor signaling pathway**	GO:0090090	11.8833	22	0.003849	up
Gene Ontology	GTPase activity	GO:0003924	81.6496	107	0.004188	up
Gene Ontology	positive regulation of cell communication	GO:0010647	160.999	196	0.004315	up
Gene Ontology	tube morphogenesis	GO:0035239	96.5995	124	0.00432	up
Gene Ontology	positive regulation of signaling pathway	GO:0035468	148.349	182	0.00432	up
Gene Ontology	endopeptidase inhibitor activity	GO:0004866	57.4997	37	0.004336	down
Gene Ontology	transmembrane receptor protein serine/threonine kinase activity	GO:0004675	6.51663	14	0.004352	up
Gene Ontology	transforming growth factor beta receptor activity	GO:0005024	6.51663	14	0.004352	up
Gene Ontology	face development	GO:0060324	6.51663	14	0.004352	up
Gene Ontology	endopeptidase regulator activity	GO:0061135	58.6497	38	0.004456	down
Gene Ontology	glucose import	GO:0046323	13.4166	24	0.00452	up
Gene Ontology	substrate-specific transporter activity	GO:0022892	369.532	421	0.004573	up
Gene Ontology	smooth muscle cell migration	GO:0014909	9.19996	18	0.004679	up
Gene Ontology	actin binding	GO:0003779	126.116	157	0.004698	up
Gene Ontology	negative regulation of cell proliferation	GO:0008285	146.816	180	0.004737	up
Gene Ontology	cytosolic small ribosomal subunit	GO:0022627	13.7999	4	0.004751	down
Gene Ontology	cellular response to insulin stimulus	GO:0032869	35.2665	52	0.004754	up
Gene Ontology	regulation of catalytic activity	GO:0050790	351.898	402	0.004754	up
Gene Ontology	cell adhesion molecule binding	GO:0050839	14.1833	25	0.004754	up
Gene Ontology	response to cytokine stimulus	GO:0034097	41.0165	59	0.004764	up
Gene Ontology	microtubule binding	GO:0008017	26.4499	41	0.004852	up
Gene Ontology	proteinaceous extracellular matrix	GO:0005578	119.983	150	0.004897	up
Gene Ontology	embryonic limb morphogenesis	GO:0030326	34.4998	51	0.004918	up
Gene Ontology	embryonic appendage morphogenesis	GO:0035113	34.4998	51	0.004918	up
Gene Ontology	cellular_component	GO:0005575	6283.19	6343	0.005033	up
Gene Ontology	mammary gland development	GO:0030879	33.7332	50	0.005109	up
Gene Ontology	regulation of neuron apoptosis	GO:0043523	33.7332	50	0.005109	up
Gene Ontology	T cell activation	GO:0042110	75.133	99	0.005144	up
Gene Ontology	regulation of protein kinase activity	GO:0045859	145.283	178	0.005161	up
Gene Ontology	regulation of cellular component size	GO:0032535	134.549	166	0.005336	up
Gene Ontology	actin filament bundle assembly	GO:0051017	18.0166	30	0.005413	up
Gene Ontology	**regulation of canonical Wnt receptor signaling pathway**	GO:0060828	18.0166	30	0.005413	up
Gene Ontology	regulation of cell-substrate	GO:0010810	21.0832	34	0.005417	up

	adhesion					
Gene Ontology	primary active transmembrane transporter activity	GO:0015399	43.6998	62	0.005441	up
Gene Ontology	P-P-bond-hydrolysis-driven transmembrane transporter activity	GO:0015405	43.6998	62	0.005441	up
Gene Ontology	cellular respiration	GO:0045333	38.3331	22	0.005583	down
Gene Ontology	transcription factor binding	GO:0008134	201.249	239	0.005895	up
Gene Ontology	hemopoietic or lymphoid organ development	GO:0048534	130.333	161	0.005939	up
Gene Ontology	negative regulation of metabolic process	GO:0009892	340.782	389	0.006097	up
Gene Ontology	signal transducer activity	GO:0004871	815.729	745	0.006118	down
Gene Ontology	molecular transducer activity	GO:0060089	815.729	745	0.006118	down
Gene Ontology	regulation of nervous system development	GO:0051960	91.2329	117	0.006133	up
Gene Ontology	nucleoplasm part	GO:0044451	229.999	270	0.006216	up
Gene Ontology	extracellular matrix	GO:0031012	142.216	174	0.006306	up
Gene Ontology	regulation of anatomical structure size	GO:0090066	159.466	193	0.006372	up
Gene Ontology	multicellular organismal process	GO:0032501	1774.44	1870	0.006463	up
Gene Ontology	mitochondrial electron transport, NADH to ubiquinone	GO:0006120	16.4833	6	0.006473	down
Gene Ontology	negative regulation of gene-specific transcription	GO:0032582	34.8832	51	0.00671	up
Gene Ontology	nucleosome	GO:0000786	24.9165	12	0.006762	down
Gene Ontology	positive regulation of cellular component movement	GO:0051272	53.2831	73	0.006791	up
Gene Ontology	regulation of glucose import	GO:0046324	12.2666	22	0.007069	up
Gene Ontology	lipid localization	GO:0010876	70.533	93	0.007076	up
Gene Ontology	regulation of smooth muscle cell migration	GO:0014910	8.04996	16	0.007076	up
Gene Ontology	protein tyrosine phosphatase activity	GO:0004725	39.0998	56	0.007214	up
Gene Ontology	Golgi stack	GO:0005795	39.0998	56	0.007214	up
Gene Ontology	regulation of T-helper cell differentiation	GO:0045622	4.21665	10	0.007242	up
Gene Ontology	prostate glandular acinus development	GO:0060525	4.21665	10	0.007242	up
Gene Ontology	lymphocyte activation	GO:0046649	108.483	136	0.007305	up
Gene Ontology	hepaticobiliary system development	GO:0061008	25.2999	39	0.007305	up
Gene Ontology	protein complex binding	GO:0032403	94.2995	120	0.007374	up
Gene Ontology	regulation of gene-specific	GO:0032583	85.4829	110	0.007374	up

	transcription					
Gene Ontology	immune system development	GO:0002520	139.149	170	0.007662	up
Gene Ontology	regulation of transferase activity	GO:0051338	156.399	189	0.0077	up
Gene Ontology	neuron migration	GO:0001764	22.2332	35	0.008042	up
Gene Ontology	extracellular matrix structural constituent	GO:0005201	31.0498	46	0.008171	up
Gene Ontology	organelle subcompartment	GO:0031984	31.0498	46	0.008171	up
Gene Ontology	regulation of neurogenesis	GO:0050767	83.9496	108	0.008244	up
Gene Ontology	cytokinesis	GO:0000910	20.6999	33	0.008268	up
Gene Ontology	proximal/distal pattern formation	GO:0009954	8.81662	17	0.008268	up
Gene Ontology	ion transport	GO:0006811	317.782	363	0.008297	up
Gene Ontology	lateral plasma membrane	GO:0016328	6.1333	13	0.008297	up
Gene Ontology	prostate gland development	GO:0030850	16.0999	27	0.008297	up
Gene Ontology	hippo signaling cascade	GO:0035329	6.1333	13	0.008297	up
Gene Ontology	SH3/SH2 adaptor activity	GO:0005070	19.1666	31	0.008376	up
Gene Ontology	coated pit	GO:0005905	18.3999	30	0.008376	up
Gene Ontology	hemopoiesis	GO:0030097	122.283	151	0.008376	up
Gene Ontology	Golgi cisterna	GO:0031985	30.2832	45	0.008376	up
Gene Ontology	appendage development	GO:0048736	41.0165	58	0.00851	up
Gene Ontology	limb development	GO:0060173	41.0165	58	0.00851	up
Gene Ontology	phosphatase activity	GO:0016791	97.3662	123	0.008741	up
Gene Ontology	clathrin-coated vesicle	GO:0030136	56.3497	76	0.009089	up
Gene Ontology	development of primary sexual characteristics	GO:0045137	56.3497	76	0.009089	up
Gene Ontology	cell projection membrane	GO:0031253	44.4664	62	0.00913	up
Gene Ontology	gastrulation with mouth forming second	GO:0001702	9.58329	18	0.00928	up
Gene Ontology	oxidative phosphorylation	GO:0006119	39.8665	24	0.009382	down
Gene Ontology	pyrophosphatase activity	GO:0016462	292.099	335	0.009382	up
Gene Ontology	subsynaptic reticulum	GO:0071212	277.149	319	0.009382	up
Gene Ontology	positive regulation of binding	GO:0051099	43.6998	61	0.009581	up
Gene Ontology	axon part	GO:0033267	27.2165	41	0.009667	up
Gene Ontology	positive regulation of transcription factor activity	GO:0051091	33.7332	49	0.009681	up
Gene Ontology	hydrolase activity, acting on acid anhydrides, in phosphorus-containing anhydrides	GO:0016818	293.249	336	0.009864	up
Gene Ontology	Rho protein signal transduction	GO:0007266	47.1498	65	0.010054	up
Gene Ontology	extracellular matrix part	GO:0044420	42.9331	60	0.010054	up
Gene Ontology	negative regulation of cell differentiation	GO:0045596	92.3829	117	0.010114	up
Gene Ontology	central nervous system neuron development	GO:0021954	10.3499	19	0.010164	up

Gene Ontology	cytosolic part	GO:0044445	56.7331	38	0.010407	down
Gene Ontology	positive regulation of gene-specific transcription from RNA polymerase II promoter	GO:0010552	42.1665	59	0.01055	up
Gene Ontology	small molecule metabolic process	GO:0044281	650.897	712	0.01055	up
Gene Ontology	liver development	GO:0001889	24.9165	38	0.010626	up
Gene Ontology	nucleoside phosphate metabolic process	GO:0006753	213.516	250	0.010688	up
Gene Ontology	nucleotide metabolic process	GO:0009117	213.516	250	0.010688	up
Gene Ontology	lymphocyte activation involved in immune response	GO:0002285	11.1166	20	0.010861	up
Gene Ontology	positive regulation of cell proliferation	GO:0008284	176.716	210	0.010861	up
Gene Ontology	negative regulation of phosphorus metabolic process	GO:0010563	24.1499	37	0.010861	up
Gene Ontology	symporter activity	GO:0015293	49.8331	68	0.010861	up
Gene Ontology	negative regulation of phosphate metabolic process	GO:0045936	24.1499	37	0.010861	up
Gene Ontology	GTPase activator activity	GO:0005096	88.1662	112	0.011019	up
Gene Ontology	receptor signaling protein serine/threonine kinase activity	GO:0004702	23.3832	36	0.011183	up
Gene Ontology	hydrolase activity, acting on acid anhydrides	GO:0016817	294.782	337	0.011245	up
Gene Ontology	neuronal cell body	GO:0043025	69.7663	91	0.011352	up
Gene Ontology	cell body	GO:0044297	69.7663	91	0.011352	up
Gene Ontology	central nervous system neuron axonogenesis	GO:0021955	4.98331	11	0.011437	up
Gene Ontology	cortical cytoskeleton organization	GO:0030865	4.98331	11	0.011437	up
Gene Ontology	cortical actin cytoskeleton organization	GO:0030866	4.98331	11	0.011437	up
Gene Ontology	eye development	GO:0001654	59.4164	79	0.011768	up
Gene Ontology	protein N-terminus binding	GO:0047485	29.8999	44	0.01178	up
Gene Ontology	cofactor catabolic process	GO:0051187	12.6499	4	0.011824	down
Gene Ontology	filopodium	GO:0030175	12.6499	22	0.011938	up
Gene Ontology	ATPase activity, coupled to movement of substances	GO:0043492	39.8665	56	0.012015	up
Gene Ontology	negative regulation of ligase activity	GO:0051352	25.2999	13	0.012015	down
Gene Ontology	negative regulation of ubiquitin-protein ligase activity	GO:0051444	25.2999	13	0.012015	down
Gene Ontology	ovulation cycle process	GO:0022602	29.1332	43	0.012191	up
Gene Ontology	regulation of endocytosis	GO:0030100	29.1332	43	0.012191	up
Gene Ontology	collagen	GO:0005581	13.4166	23	0.012255	up
Gene Ontology	response to bacterium	GO:0009617	91.2329	68	0.012402	down

Gene Ontology	SNAP receptor activity	GO:0005484	7.66663	15	0.012435	up
Gene Ontology	RNA splicing	GO:0008380	114.999	89	0.01254	down
Gene Ontology	appendage morphogenesis	GO:0035107	39.0998	55	0.01254	up
Gene Ontology	limb morphogenesis	GO:0035108	39.0998	55	0.01254	up
Gene Ontology	presynaptic membrane	GO:0042734	14.9499	25	0.012565	up
Gene Ontology	dendritic spine	GO:0043197	18.0166	29	0.012565	up
Gene Ontology	neuron spine	GO:0044309	18.0166	29	0.012565	up
Gene Ontology	recycling endosome	GO:0055037	14.9499	25	0.012565	up
Gene Ontology	positive regulation of transcription regulator activity	GO:0090047	34.1165	49	0.012565	up
Gene Ontology	vasculogenesis	GO:0001570	16.4833	27	0.012656	up
Gene Ontology	sarcoplasmic reticulum	GO:0016529	16.4833	27	0.012656	up
Gene Ontology	intrinsic to organelle membrane	GO:0031300	57.8831	77	0.012817	up
Gene Ontology	regulation of cellular protein metabolic process	GO:0032268	220.799	257	0.01289	up
Gene Ontology	protein amino acid glycosylation	GO:0006486	46.7664	64	0.012938	up
Gene Ontology	multicellular organism growth	GO:0035264	27.5999	41	0.012938	up
Gene Ontology	macromolecule glycosylation	GO:0043413	46.7664	64	0.012938	up
Gene Ontology	glycosylation	GO:0070085	46.7664	64	0.012938	up
Gene Ontology	neuron death	GO:0070997	38.3331	54	0.012956	up
Gene Ontology	transmembrane receptor protein serine/threonine kinase signaling pathway	GO:0007178	64.0164	84	0.013191	up
Gene Ontology	positive regulation of programmed cell death	GO:0043068	179.399	212	0.013594	up
Gene Ontology	**cell-cell adhesion**	GO:0016337	121.899	149	0.0136	up
Gene Ontology	positive regulation of DNA binding	GO:0043388	37.5665	53	0.0136	up
Gene Ontology	neuron apoptosis	GO:0051402	37.5665	53	0.0136	up
Gene Ontology	positive regulation of cell death	GO:0010942	181.316	214	0.013855	up
Gene Ontology	integral to organelle membrane	GO:0031301	49.4498	67	0.013947	up
Gene Ontology	regulation of cell projection assembly	GO:0060491	8.43329	16	0.013956	up
Gene Ontology	nucleoplasm	GO:0005654	359.948	405	0.014029	up
Gene Ontology	3',5'-cyclic-AMP phosphodiesterase activity	GO:0004115	3.83331	9	0.014438	up
Gene Ontology	asymmetric protein localization	GO:0008105	3.83331	9	0.014438	up
Gene Ontology	Cul3-RING ubiquitin ligase complex	GO:0031463	3.83331	9	0.014438	up
Gene Ontology	I-SMAD binding	GO:0070411	3.83331	9	0.014438	up
Gene Ontology	regulation of growth	GO:0040008	142.983	172	0.01452	up
Gene Ontology	coated vesicle	GO:0030135	68.6163	89	0.01478	up
Gene Ontology	positive regulation of protein ubiquitination	GO:0031398	36.4165	22	0.01478	down
Gene Ontology	cation channel complex	GO:0034703	52.1331	70	0.014802	up

Gene Ontology	integrin binding	GO:0005178	24.5332	37	0.014831	up
Gene Ontology	positive regulation of microtubule polymerization	GO:0031116	2.68332	7	0.014867	up
Gene Ontology	nucleobase, nucleoside and nucleotide metabolic process	GO:0055086	222.332	258	0.014889	up
Gene Ontology	CD4-positive, alpha-beta T cell differentiation involved in immune response	GO:0002294	5.74997	12	0.015028	up
Gene Ontology	phospholipid-translocating ATPase activity	GO:0004012	5.74997	12	0.015028	up
Gene Ontology	hydrolase activity, acting on acid anhydrides, catalyzing transmembrane movement of substances	GO:0016820	40.2498	56	0.015028	up
Gene Ontology	T-helper cell differentiation	GO:0042093	5.74997	12	0.015028	up
Gene Ontology	glycosphingolipid metabolic process	GO:0006687	9.19996	17	0.015283	up
Gene Ontology	receptor binding	GO:0005102	355.732	400	0.015413	up
Gene Ontology	positive regulation of kinase activity	GO:0033674	96.2162	120	0.015659	up
Gene Ontology	ATPase activity, coupled to transmembrane movement of substances	GO:0042626	39.4831	55	0.015811	up
Gene Ontology	positive regulation of apoptosis	GO:0043065	178.249	210	0.016465	up
Gene Ontology	response to stress	GO:0006950	730.246	791	0.016587	up
Gene Ontology	intracellular protein transport	GO:0006886	168.283	199	0.017349	up
Gene Ontology	insulin receptor signaling pathway	GO:0008286	20.6999	32	0.017349	up
Gene Ontology	stress fiber	GO:0001725	10.7333	19	0.017608	up
Gene Ontology	amine transmembrane transporter activity	GO:0005275	27.9832	41	0.017608	up
Gene Ontology	striated muscle tissue development	GO:0014706	62.8664	82	0.017748	up
Gene Ontology	positive regulation of ubiquitin-protein ligase activity	GO:0051443	27.2165	15	0.017751	down
Gene Ontology	glycogen metabolic process	GO:0005977	19.1666	30	0.018222	up
Gene Ontology	potassium ion transport	GO:0006813	52.5164	70	0.018344	up
Gene Ontology	regulation of BMP signaling pathway	GO:0030510	11.4999	20	0.018344	up
Gene Ontology	response to insulin stimulus	GO:0032868	52.5164	70	0.018344	up
Gene Ontology	leukocyte differentiation	GO:0002521	74.3663	95	0.018411	up
Gene Ontology	axon guidance	GO:0007411	37.1832	52	0.018411	up
Gene Ontology	lipid transport	GO:0006869	62.0997	81	0.018654	up
Gene Ontology	translation	GO:0006412	157.933	129	0.018749	down
Gene Ontology	developmental pigmentation	GO:0048066	12.2666	21	0.018806	up

Gene Ontology	cytosol	GO:0005829	507.148	558	0.018888	up
Gene Ontology	histone deacetylase complex	GO:0000118	16.8666	27	0.018889	up
Gene Ontology	negative regulation of protein amino acid phosphorylation	GO:0001933	16.8666	27	0.018889	up
Gene Ontology	sarcoplasm	GO:0016528	16.8666	27	0.018889	up
Gene Ontology	Golgi cisterna membrane	GO:0032580	26.4499	39	0.018889	up
Gene Ontology	ATP synthesis coupled electron transport	GO:0042773	21.8499	11	0.018889	down
Gene Ontology	mitochondrial ATP synthesis coupled electron transport	GO:0042775	21.8499	11	0.018889	down
Gene Ontology	positive regulation of ligase activity	GO:0051351	28.3665	16	0.018889	down
Gene Ontology	regulation of response to stress	GO:0080134	126.499	153	0.018889	up
Gene Ontology	cartilage development	GO:0051216	36.4165	51	0.019116	up
Gene Ontology	killing of cells of another organism	GO:0031640	8.81662	2	0.019255	down
Gene Ontology	transporter activity	GO:0005215	450.031	498	0.019292	up
Gene Ontology	regulation of intracellular protein kinase cascade	GO:0010627	128.416	155	0.019324	up
Gene Ontology	induction of programmed cell death	GO:0012502	128.416	155	0.019324	up
Gene Ontology	cellular component assembly	GO:0022607	398.665	444	0.019355	up
Gene Ontology	positive regulation of cell projection organization	GO:0031346	25.6832	38	0.019465	up
Gene Ontology	melanosome	GO:0042470	35.6498	50	0.019944	up
Gene Ontology	cellular lipid metabolic process	GO:0044255	225.399	260	0.019944	up
Gene Ontology	pigment granule	GO:0048770	35.6498	50	0.019944	up
Gene Ontology	negative regulation of developmental process	GO:0051093	113.083	138	0.019944	up
Gene Ontology	ion transmembrane transporter activity	GO:0015075	274.082	312	0.019951	up
Gene Ontology	peptidase inhibitor activity	GO:0030414	60.9497	43	0.019961	down
Gene Ontology	myeloid cell differentiation	GO:0030099	60.5664	79	0.020336	up
Gene Ontology	positive regulation of molecular function	GO:0044093	249.932	286	0.021101	up
Gene Ontology	regulation of protein metabolic process	GO:0051246	249.932	286	0.021101	up
Gene Ontology	sex determination	GO:0007530	7.2833	14	0.021374	up
Gene Ontology	melanocyte differentiation	GO:0030318	7.2833	14	0.021374	up
Gene Ontology	regulation of developmental pigmentation	GO:0048070	4.59998	10	0.021571	up
Gene Ontology	positive regulation of gene-specific transcription	GO:0043193	56.3497	74	0.02192	up
Gene Ontology	small GTPase regulator activity	GO:0005083	111.549	136	0.022001	up
Gene Ontology	gastrulation	GO:0007369	34.1165	48	0.022008	up

Gene Ontology	embryonic organ morphogenesis	GO:0048562	52.8997	70	0.022124	up
Gene Ontology	regulation of cell shape	GO:0008360	22.6166	34	0.022573	up
Gene Ontology	amino acid transmembrane transporter activity	GO:0015171	22.6166	34	0.022573	up
Gene Ontology	protein heterooligomerization	GO:0051291	22.6166	34	0.022573	up
Gene Ontology	transmembrane transport	GO:0055085	233.449	268	0.022925	up
Gene Ontology	induction of apoptosis	GO:0006917	128.033	154	0.022929	up
Gene Ontology	solute:cation symporter activity	GO:0015294	33.3498	47	0.023029	up
Gene Ontology	female sex differentiation	GO:0046660	33.3498	47	0.023029	up
Gene Ontology	regulation of myeloid cell differentiation	GO:0045637	28.3665	41	0.023099	up
Gene Ontology	chemical homeostasis	GO:0048878	215.816	249	0.023437	up
Gene Ontology	protein dimerization activity	GO:0046983	226.166	260	0.023992	up
Gene Ontology	ATPase activity, coupled to transmembrane movement of ions, phosphorylative mechanism	GO:0015662	20.3166	31	0.025113	up
Gene Ontology	cellular glucan metabolic process	GO:0006073	19.5499	30	0.02587	up
Gene Ontology	carbohydrate transport	GO:0008643	36.0332	50	0.02587	up
Gene Ontology	synaptosome	GO:0019717	36.0332	50	0.02587	up
Gene Ontology	glucan metabolic process	GO:0044042	19.5499	30	0.02587	up
Gene Ontology	head development	GO:0060322	8.81662	16	0.02587	up
Gene Ontology	fertilization	GO:0009566	30.2832	18	0.026373	down
Gene Ontology	cell cortex part	GO:0044448	26.0665	38	0.026577	up
Gene Ontology	negative regulation of neuron apoptosis	GO:0043524	18.7832	29	0.02661	up
Gene Ontology	extracellular matrix organization	GO:0030198	39.4831	54	0.02681	up
Gene Ontology	proton-transporting ATP synthase complex	GO:0045259	8.43329	2	0.026966	down
Gene Ontology	positive regulation of locomotion	GO:0040017	53.2831	70	0.027156	up
Gene Ontology	cofactor biosynthetic process	GO:0051188	42.9331	58	0.027345	up
Gene Ontology	digestive tract morphogenesis	GO:0048546	9.58329	17	0.027352	up
Gene Ontology	positive regulation of cell migration	GO:0030335	49.8331	66	0.027405	up
Gene Ontology	protein serine/threonine phosphatase activity	GO:0004722	17.2499	27	0.027961	up
Gene Ontology	response to carbohydrate stimulus	GO:0009743	30.2832	43	0.028065	up
Gene Ontology	paraxial mesoderm development	GO:0048339	5.36664	11	0.028065	up
Gene Ontology	lipid modification	GO:0030258	38.7165	53	0.028129	up
Gene Ontology	cation transmembrane transporter activity	GO:0008324	210.066	242	0.028325	up
Gene Ontology	nerve development	GO:0021675	10.3499	18	0.028325	up
Gene Ontology	cellular monovalent inorganic cation homeostasis	GO:0030004	10.3499	18	0.028325	up
Gene Ontology	synaptic vesicle membrane	GO:0030672	16.4833	26	0.028325	up
Gene Ontology	regulation of Ras protein signal	GO:0046578	89.6996	111	0.028325	up

	transduction					
Gene Ontology	protein amino acid autophosphorylation	GO:0046777	34.4998	48	0.028325	up
Gene Ontology	nuclear lumen	GO:0031981	581.897	633	0.028608	up
Gene Ontology	digestive system development	GO:0055123	24.5332	36	0.028608	up
Gene Ontology	regulation of protein localization	GO:0032880	67.4663	86	0.028852	up
Gene Ontology	mitochondrial membrane organization	GO:0007006	11.1166	19	0.02897	up
Gene Ontology	foregut morphogenesis	GO:0007440	3.44998	8	0.028975	up
Gene Ontology	coenzyme biosynthetic process	GO:0009108	29.5165	42	0.028975	up
Gene Ontology	cyclic nucleotide catabolic process	GO:0009214	3.44998	8	0.028975	up
Gene Ontology	fucose catabolic process	GO:0019317	3.44998	8	0.028975	up
Gene Ontology	L-fucose catabolic process	GO:0042355	3.44998	8	0.028975	up
Gene Ontology	regulation of cytoskeleton organization	GO:0051493	55.1997	72	0.029167	up
Gene Ontology	regulation of cyclase activity	GO:0031279	37.9498	52	0.029186	up
Gene Ontology	regulation of cell-matrix adhesion	GO:0001952	13.4166	22	0.029192	up
Gene Ontology	insulin receptor binding	GO:0005158	11.8833	20	0.029192	up
Gene Ontology	protein amino acid N-linked glycosylation	GO:0006487	13.4166	22	0.029192	up
Gene Ontology	regulation of glucose transport	GO:0010827	13.4166	22	0.029192	up
Gene Ontology	actomyosin	GO:0042641	12.6499	21	0.029192	up
Gene Ontology	regulation of fatty acid oxidation	GO:0046320	12.6499	21	0.029192	up
Gene Ontology	synaptic vesicle transport	GO:0048489	12.6499	21	0.029192	up
Gene Ontology	phosphoprotein binding	GO:0051219	12.6499	21	0.029192	up
Gene Ontology	regulation of immune system process	GO:0002682	162.916	191	0.029463	up
Gene Ontology	regulation of protein modification process	GO:0031399	144.516	171	0.029792	up
Gene Ontology	response to estrogen stimulus	GO:0043627	44.8498	60	0.029792	up
Gene Ontology	respiratory electron transport chain	GO:0022904	24.9165	14	0.03036	down
Gene Ontology	voltage-gated potassium channel activity	GO:0005249	37.1832	51	0.030466	up
Gene Ontology	Ras guanyl-nucleotide exchange factor activity	GO:0005088	32.9665	46	0.030719	up
Gene Ontology	protein oligomerization	GO:0051259	74.7496	94	0.030719	up
Gene Ontology	energy reserve metabolic process	GO:0006112	22.2332	33	0.031866	up
Gene Ontology	T cell cytokine production	GO:0002369	2.29999	6	0.031969	up
Gene Ontology	NF-kappaB-inducing kinase activity	GO:0004704	2.29999	6	0.031969	up
Gene Ontology	FACIT collagen	GO:0005593	2.29999	6	0.031969	up
Gene Ontology	protein serine/threonine kinase activator activity	GO:0043539	2.29999	6	0.031969	up

Gene Ontology	regulation of T-helper 2 cell differentiation	GO:0045628	2.29999	6	0.031969	up
Gene Ontology	positive regulation of protein kinase activity	GO:0045860	92.7662	114	0.031969	up
Gene Ontology	positive regulation of focal adhesion assembly	GO:0051894	2.29999	6	0.031969	up
Gene Ontology	prostate glandular acinus morphogenesis	GO:0060526	2.29999	6	0.031969	up
Gene Ontology	prostate epithelial cord arborization involved in prostate glandular acinus morphogenesis	GO:0060527	2.29999	6	0.031969	up
Gene Ontology	cellular response to transforming growth factor beta stimulus	GO:0071560	2.29999	6	0.031969	up
Gene Ontology	RNA splicing, via transesterification reactions with bulged adenosine as nucleophile	GO:0000377	37.1832	24	0.03209	down
Gene Ontology	nuclear mRNA splicing, via spliceosome	GO:0000398	37.1832	24	0.03209	down
Gene Ontology	alpha-beta T cell activation involved in immune response	GO:0002287	6.1333	12	0.03209	up
Gene Ontology	T cell differentiation involved in immune response	GO:0002292	6.1333	12	0.03209	up
Gene Ontology	alpha-beta T cell differentiation involved in immune response	GO:0002293	6.1333	12	0.03209	up
Gene Ontology	**cell-cell junction**	GO:0005911	76.6663	96	0.03209	up
Gene Ontology	regulation of glycogen biosynthetic process	GO:0005979	6.1333	12	0.03209	up
Gene Ontology	regulation of glucan biosynthetic process	GO:0010962	6.1333	12	0.03209	up
Gene Ontology	regulation of polysaccharide biosynthetic process	GO:0032885	6.1333	12	0.03209	up
Gene Ontology	myelin sheath	GO:0043209	6.1333	12	0.03209	up
Gene Ontology	regulation of response to stimulus	GO:0048583	208.916	240	0.032594	up
Gene Ontology	leukocyte activation	GO:0045321	131.099	156	0.032636	up
Gene Ontology	protein kinase binding	GO:0019901	67.8497	86	0.033062	up
Gene Ontology	development of primary female sexual characteristics	GO:0046545	31.4332	44	0.033343	up
Gene Ontology	eye morphogenesis	GO:0048592	31.4332	44	0.033343	up
Gene Ontology	cation transport	GO:0006812	225.016	257	0.034174	up
Gene Ontology	cysteine-type peptidase activity	GO:0008234	55.5831	72	0.034344	up
Gene Ontology	late endosome	GO:0005770	42.5498	57	0.034393	up
Gene Ontology	RNA splicing, via transesterification reactions	GO:0000375	40.6331	27	0.034706	down
Gene Ontology	caveola	GO:0005901	19.9332	30	0.034945	up

Gene Ontology	positive regulation of organelle organization	GO:0010638	34.8832	48	0.034945	up
Gene Ontology	sphingolipid metabolic process	GO:0006665	30.6665	43	0.035036	up
Gene Ontology	small conjugating protein ligase activity	GO:0019787	80.4996	100	0.035397	up
Gene Ontology	negative regulation of gene-specific transcription from RNA polymerase II promoter	GO:0010553	25.6832	37	0.035509	up
Gene Ontology	viral infectious cycle	GO:0019058	25.6832	37	0.035509	up
Gene Ontology	developmental maturation	GO:0021700	38.3331	52	0.035796	up
Gene Ontology	threonine-type endopeptidase activity	GO:0004298	8.04996	2	0.035942	down
Gene Ontology	threonine-type peptidase activity	GO:0070003	8.04996	2	0.035942	down
Gene Ontology	actin cytoskeleton	GO:0015629	107.716	130	0.036763	up
Gene Ontology	large ribosomal subunit	GO:0015934	24.5332	14	0.036906	down
Gene Ontology	regulation of neuron differentiation	GO:0045664	68.9997	87	0.036913	up
Gene Ontology	transcription coactivator activity	GO:0003713	82.4163	102	0.037018	up
Gene Ontology	response to estradiol stimulus	GO:0032355	24.9165	36	0.037025	up
Gene Ontology	steroid dehydrogenase activity, acting on the CH-OH group of donors, NAD or NADP as acceptor	GO:0033764	9.58329	3	0.037025	down
Gene Ontology	cell junction assembly	GO:0034329	24.9165	36	0.037025	up
Gene Ontology	intrinsic to Golgi membrane	GO:0031228	18.3999	28	0.037174	up
Gene Ontology	vacuole	GO:0005773	109.633	132	0.037777	up
Gene Ontology	endoplasmic reticulum part	GO:0044432	264.115	298	0.038315	up
Gene Ontology	voltage-gated potassium channel complex	GO:0008076	33.3498	46	0.038509	up
Gene Ontology	potassium channel complex	GO:0034705	33.3498	46	0.038509	up
Gene Ontology	response to glucose stimulus	GO:0009749	24.1499	35	0.038657	up
Gene Ontology	growth cone	GO:0030426	24.1499	35	0.038657	up
Gene Ontology	cell cortex	GO:0005938	50.5998	66	0.038798	up
Gene Ontology	pigment cell differentiation	GO:0050931	7.66663	14	0.038998	up
Gene Ontology	protein-DNA complex	GO:0032993	37.9498	25	0.039068	down
Gene Ontology	columnar/cuboidal epithelial cell differentiation	GO:0002065	4.21665	9	0.039863	up
Gene Ontology	fibrillar collagen	GO:0005583	4.21665	9	0.039863	up
Gene Ontology	respiratory tube development	GO:0030323	40.2498	54	0.039863	up
Gene Ontology	actinin binding	GO:0042805	4.21665	9	0.039863	up
Gene Ontology	protein phosphatase 2A binding	GO:0051721	4.21665	9	0.039863	up
Gene Ontology	regulation of cell growth	GO:0001558	83.5663	103	0.040478	up
Gene Ontology	regulation of binding	GO:0051098	80.8829	100	0.040698	up
Gene Ontology	gonad development	GO:0008406	49.8331	65	0.040817	up
Gene Ontology	positive regulation of	GO:0002053	8.43329	15	0.041027	up

	mesenchymal cell proliferation					
Gene Ontology	lipid biosynthetic process	GO:0008610	147.583	173	0.041027	up
Gene Ontology	positive regulation of Wnt receptor signaling pathway	GO:0030177	8.43329	15	0.041027	up
Gene Ontology	cadherin binding	GO:0045296	8.43329	15	0.041027	up
Gene Ontology	platelet-derived growth factor receptor signaling pathway	GO:0048008	8.43329	15	0.041027	up
Gene Ontology	cell-substrate junction assembly	GO:0007044	15.3333	24	0.041381	up
Gene Ontology	negative regulation of phosphorylation	GO:0042326	22.6166	33	0.041897	up
Gene Ontology	positive regulation of NF-kappaB transcription factor activity	GO:0051092	22.6166	33	0.041897	up
Gene Ontology	cortical cytoskeleton	GO:0030863	14.5666	23	0.042277	up
Gene Ontology	ovulation cycle	GO:0042698	31.8165	44	0.042277	up
Gene Ontology	regulation of mesenchymal cell proliferation	GO:0010464	9.19996	16	0.042544	up
Gene Ontology	microtubule-based transport	GO:0010970	9.19996	16	0.042544	up
Gene Ontology	polysaccharide metabolic process	GO:0005976	49.0664	64	0.042738	up
Gene Ontology	metal ion transmembrane transporter activity	GO:0046873	49.0664	64	0.042738	up
Gene Ontology	kinase activator activity	GO:0019209	9.96662	17	0.043596	up
Gene Ontology	lactation	GO:0007595	10.7333	18	0.043754	up
Gene Ontology	learning or memory	GO:0007611	42.1665	56	0.043754	up
Gene Ontology	vitamin biosynthetic process	GO:0009110	12.2666	20	0.043754	up
Gene Ontology	mesenchymal cell proliferation	GO:0010463	10.7333	18	0.043754	up
Gene Ontology	regulation of cell morphogenesis involved in differentiation	GO:0010769	38.7165	52	0.043754	up
Gene Ontology	ligase activity, forming carbon-nitrogen bonds	GO:0016879	104.649	126	0.043754	up
Gene Ontology	regulation of dephosphorylation	GO:0035303	12.2666	20	0.043754	up
Gene Ontology	regulation of lyase activity	GO:0051339	38.7165	52	0.043754	up
Gene Ontology	regulation of cell cycle	GO:0051726	172.882	200	0.043754	up
Gene Ontology	actin filament bundle	GO:0032432	11.4999	19	0.043879	up
Gene Ontology	regulation of heart contraction	GO:0008016	31.0498	43	0.044011	up
Gene Ontology	proteasome complex	GO:0000502	24.1499	14	0.044458	down
Gene Ontology	single fertilization	GO:0007338	24.1499	14	0.044458	down
Gene Ontology	regulation of synaptic transmission	GO:0050804	48.2998	63	0.044712	up
Gene Ontology	digestive tract development	GO:0048565	21.0832	31	0.045133	up
Gene Ontology	telencephalon development	GO:0021537	34.4998	47	0.045422	up
Gene Ontology	induction of apoptosis by extracellular signals	GO:0008624	41.3998	55	0.046112	up
Gene Ontology	acid-amino acid ligase activity	GO:0016881	93.9162	114	0.046188	up
Gene Ontology	clathrin coated vesicle membrane	GO:0030665	30.2832	42	0.046312	up

Gene Ontology	ubiquitin-protein ligase activity	GO:0004842	73.2163	91	0.047072	up
Gene Ontology	regulation of ubiquitin-protein ligase activity	GO:0051438	31.4332	20	0.048047	down
Gene Ontology	cell activation	GO:0001775	149.116	174	0.048092	up
Gene Ontology	lipid oxidation	GO:0034440	25.2999	36	0.048139	up
Gene Ontology	negative regulation of transferase activity	GO:0051348	44.0831	58	0.048139	up
Gene Ontology	ovulation from ovarian follicle	GO:0001542	4.98331	10	0.04827	up
Gene Ontology	aging	GO:0007568	50.2164	65	0.04827	up
Gene Ontology	endosome membrane	GO:0010008	80.4996	99	0.04827	up
Gene Ontology	sodium ion transmembrane transporter activity	GO:0015081	29.5165	41	0.04827	up
Gene Ontology	GMP binding	GO:0019002	4.98331	10	0.04827	up
Gene Ontology	tumor necrosis factor-mediated signaling pathway	GO:0033209	4.98331	10	0.04827	up
Gene Ontology	regulation of CD4-positive, alpha beta T cell differentiation	GO:0043370	4.98331	10	0.04827	up
Gene Ontology	endosomal part	GO:0044440	80.4996	99	0.04827	up
Gene Ontology	regulation of adenylate cyclase activity	GO:0045761	37.1832	50	0.04827	up
Gene Ontology	negative regulation of heart contraction	GO:0045822	4.98331	10	0.04827	up
Gene Ontology	microtubule polymerization	GO:0046785	4.98331	10	0.04827	up
Gene Ontology	mitochondrial proton-transporting ATP synthase complex	GO:0005753	7.66663	2	0.048343	down
Gene Ontology	cellular macromolecule catabolic process	GO:0044265	183.616	211	0.048343	up
Gene Ontology	endoplasmic reticulum membrane	GO:0005789	238.049	269	0.048405	up
Gene Ontology	endonuclease activity, active with either ribo- or deoxyribonucleic acids and producing 3'-phosphomonoesters	GO:0016894	9.19996	3	0.048785	down
Gene Ontology	regulation of ligase activity	GO:0051340	32.5832	21	0.048785	down
Gene Ontology	nucleobase, nucleoside and nucleotide biosynthetic process	GO:0034404	114.233	136	0.049343	up
Gene Ontology	nucleobase, nucleoside, nucleotide and nucleic acid biosynthetic process	GO:0034654	114.233	136	0.049343	up
Gene Ontology	nuclear membrane-endoplasmic reticulum network	GO:0042175	243.799	275	0.049343	up
Gene Ontology	peptidyl-serine modification	GO:0018209	24.5332	35	0.0497	up
Gene Ontology	site of polarized growth	GO:0030427	24.5332	35	0.0497	up
Gene Ontology	respiratory system development	GO:0060541	43.3165	57	0.049984	up

* The list of enriched pathways ($P < 0.05$, sort by FDR) in the genes predicted to be targeted by the selected 8 microRNA. In addition to associations with more general functional KEGG or GO terms, an overrepresentation of gene targets associated with cancer was seen for the selected miRNAs. Pathways which are well known to be linked with tumor-promoting function were marked in bold.

Figures

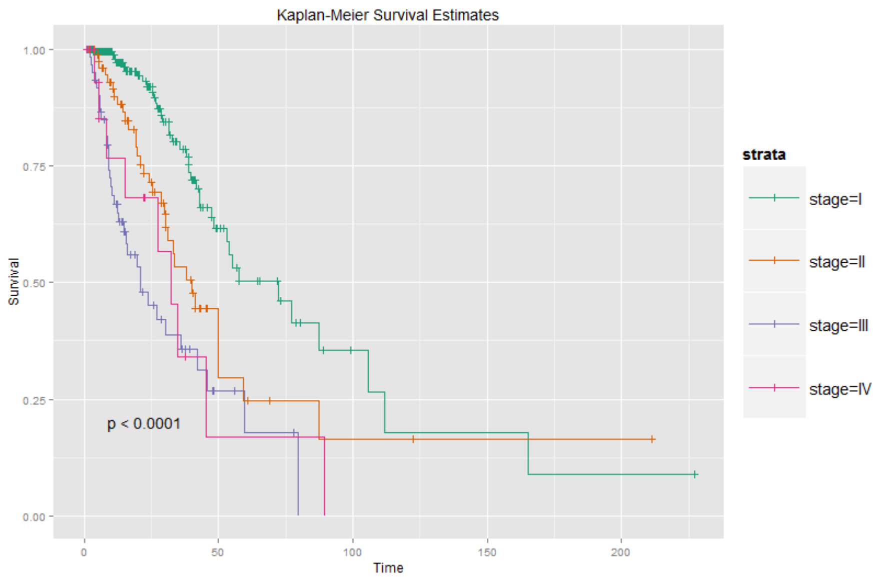

Figure S1. The prognostic role of disease stage in LUAD. Kaplan-Meier analysis of overall survival based on disease stage, n=371, 1 observations deleted due to missing values. The median OSs for stage I to IV were 72.5 months, 40.3 months, 20.9 months and 32.5 months, respectively. Patients in early stage possessed with significantly good overall survival compared with that of patients in high stage, $P < 0.001$.

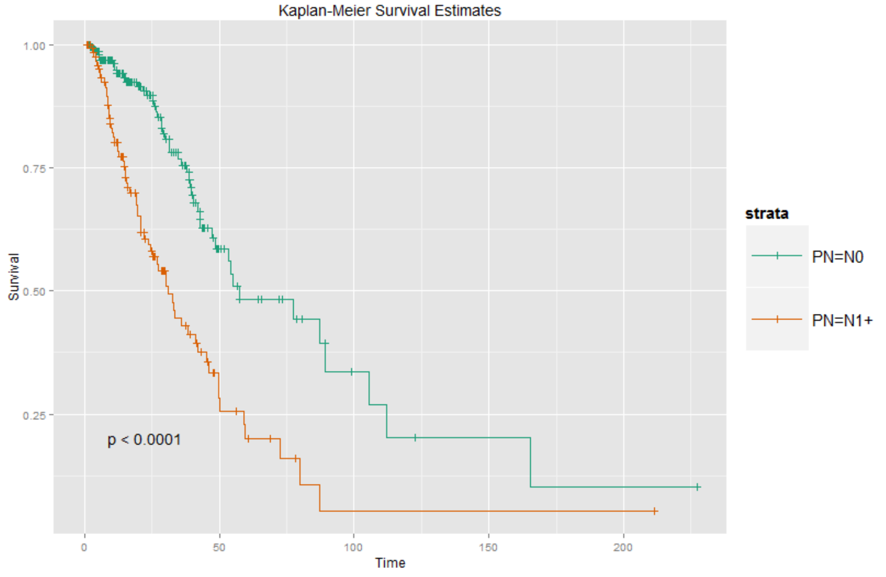

Figure S2. The prognostic role of N stage in LUAD. Kaplan-Meier analysis of overall survival based on M stage, n=362, 10 observations deleted due to missing values. Patients in N0 stage (n=231) possessed with significantly good overall survival compared with that of patients in N1+ stage (n=131) (median OS: 57.5 months *vs.* 31.0 months), $P < 0.001$.

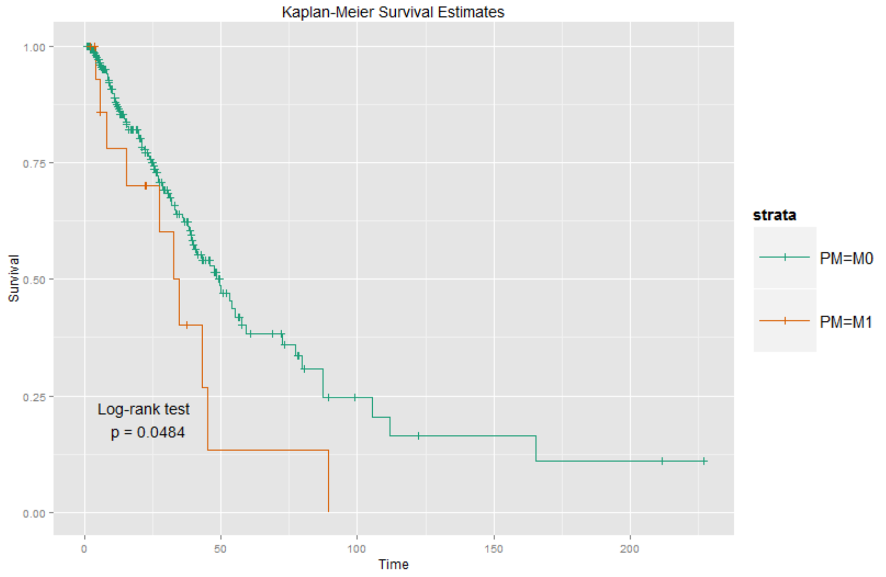

Figure S3. The prognostic role of M stage in LUAD. Kaplan-Meier analysis of overall survival based on M stage, n=282, 90 observations deleted due to missing values. Patients in M0 stage (n=266) possessed with significantly good overall survival compared with that of patients in M1 stage (n=16) (median OS: 49.7 months *vs.* 34.7 months), $P = 0.048$.

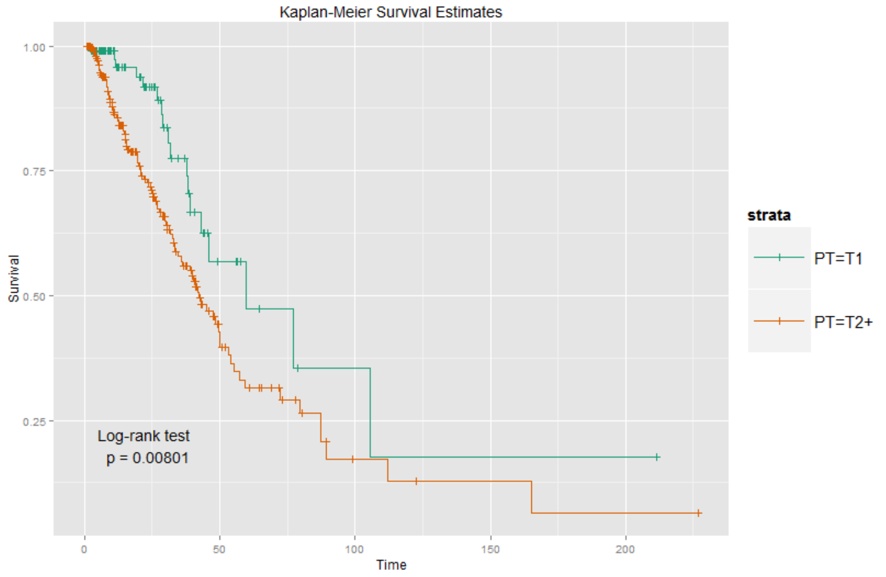

Figure S4. The prognostic role of T stage in LUAD. Kaplan-Meier analysis of overall survival based on M stage, n=370, 2 observations deleted due to missing values. Patients in T1 stage (n=112) possessed with significantly good overall survival compared with that of patients in T2+ stage (n=258) (median OS: 59.7 months *vs.* 42.2 months), $P = 0.008$.

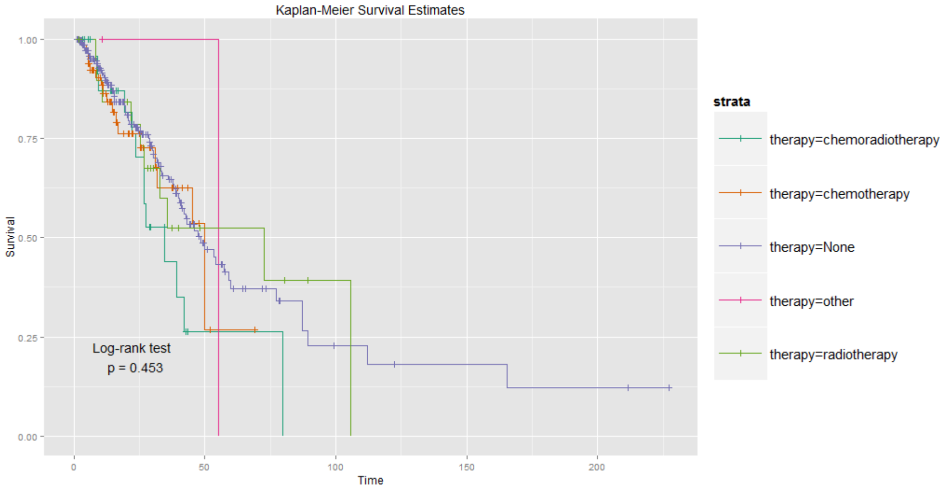

Figure S5. The prognostic role of therapy in LUAD. Kaplan-Meier analysis of OS based on therapy, no significant association was found. The median OSs for different therapy (chemotherapy, radiotherapy, chemo-radiotherapy, other and none) were 49.7 months, 72.5 months, 34.7 months, 55.1 months and 48.4 months, respectively, *P* = 0.453.

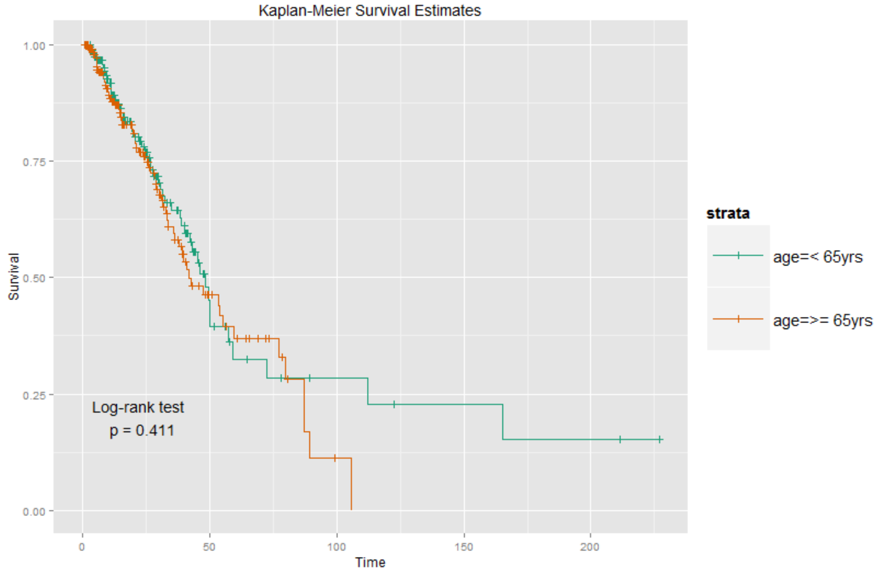

Figure S6. The prognostic role of age in LUAD. Kaplan-Meier analysis of overall survival based on age, n=372. Patients were divided into 2 groups using 65yrs for the cutoff point of age at initial pathologic diagnosis. No significant association was found between age and OS in TCGA LUAD cohort. Median OSs for young group and old group were 48.4 months and 41.9 months, respectively, *P* = 0.411.

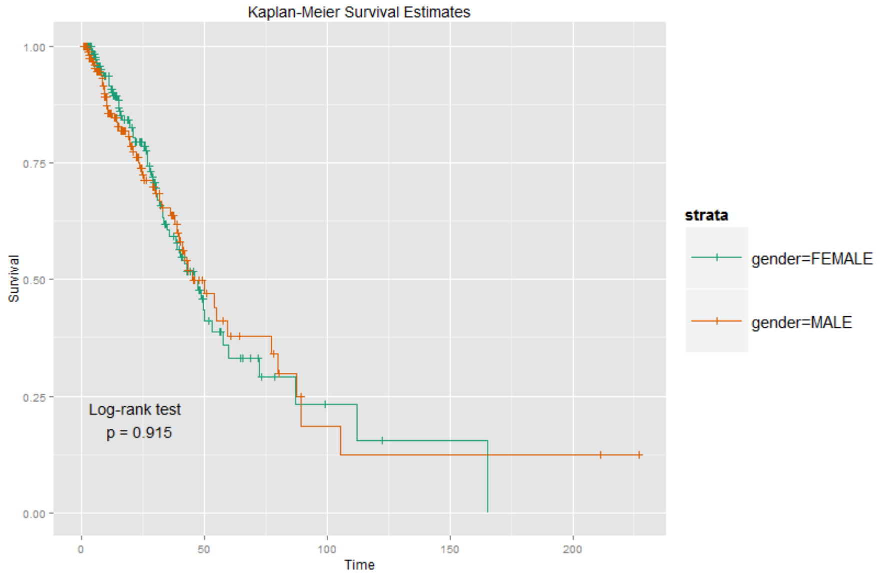

Figure S7. The prognostic role of gender in LUAD. Kaplan-Meier analysis of OS based on gender, no significant association was found. Median OSs for male and female were 45.2 months and 46.0 months, respectively, $P = 0.915$.

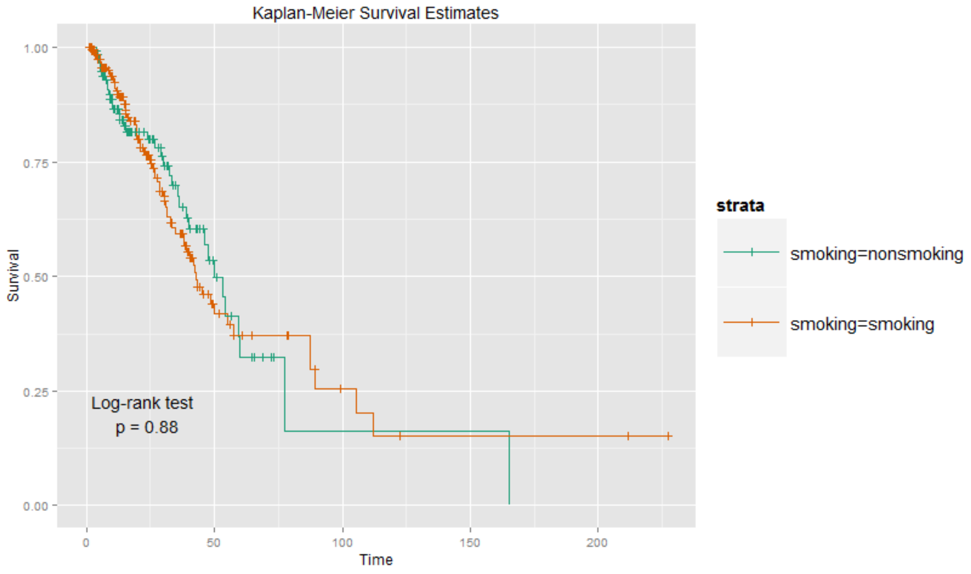

Figure S8. The prognostic role of smoking status in LUAD. Kaplan-Meier analysis of overall survival based on smoking status, no significant association was found. Median OSs for non-smoking patients and smoking patients were 49.7 months and 42.9 months, respectively, $P = 0.880$.

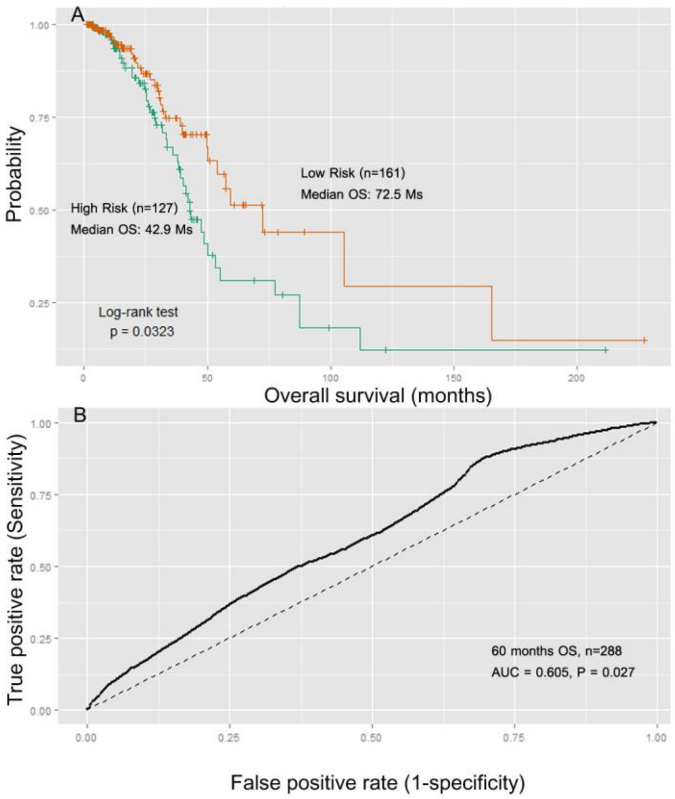

Figure S9. Kaplan–Meier and ROC curves for the 8-miRNA signature for patients of stage I and II. (A) The Kaplan–Meier curves for LUAD risk groups o for patients of stage I and II (n = 288) divided by the median cutoff point. (B) The ROC curve had an AUC of 0.605 (P = 0.027). The permutation *P* value was obtained from 1,000 permutations for testing the null hypothesis (AUC = 0.5)
